# Do evidence summaries increase health policy‐makers' use of evidence from systematic reviews? A systematic review

**DOI:** 10.4073/csr.2018.8

**Published:** 2018-09-10

**Authors:** Jennifer Petkovic, Vivian Welch, Marie Helena Jacob, Manosila Yoganathan, Ana Patricia Ayala, Heather Cunningham, Peter Tugwell

## Abstract

This review summarizes the evidence from six randomized controlled trials that judged the effectiveness of systematic review summaries on policymakers' decision making, or the most effective ways to present evidence summaries to increase policymakers' use of the evidence.

This review included six randomized controlled studies. A randomized controlled study is one in which the participants are divided randomly (by chance) into separate groups to compare different treatments or other interventions. This method of dividing people into groups means that the groups will be similar and that the effects of the treatments they receive will be compared more fairly. At the time the study is done, it is not known which treatment is the better one.

The researchers who did these studies invited people from Europe, North America, South America, Africa, and Asia to take part in them. Two studies looked at “policy briefs,” one study looked at an “evidence summary,” two looked at a “summary of findings table,” and one compared a “summary of findings table” to an evidence summary.

None of these studies looked at how policymakers directly used evidence from systematic reviews in their decision making, but two studies found that there was little to no difference in how they used the summaries. The studies relied on reports from decision makers. These studies included questions such as, “Is this summary easy to understand?”

Some of the studies looked at users' knowledge, understanding, beliefs, or how credible (trustworthy) they believed the summaries to be. There was little to no difference in the studies that looked at these outcomes. Study participants rated the graded entry format higher for usability than the full systematic review. The graded entry format allows the reader to select how much information they want to read. The study participants felt that all evidence summary formats were easier to understand than full systematic reviews.

**Plain language summary:**

**Policy briefs make systematic reviews easier to understand but little evidence of impact on use of study findings**

It is likely that evidence summaries are easier to understand than complete systematic reviews. Whether these summaries increase the use of evidence from systematic reviews in policymaking is not clear.

**What is this review about?**

Systematic reviews are long and technical documents that may be hard for policymakers to use when making decisions. Evidence summaries are short documents that describe research findings in systematic reviews. These summaries may simplify the use of systematic reviews.

Other names for evidence reviews are *policy briefs*, *evidence briefs*, *summaries of findings*, or *plain language summaries*. The goal of this review was to learn whether evidence summaries help policymakers use evidence from systematic reviews. This review also aimed to identify the best ways to present the evidence summary to increase the use of evidence.

**What is the aim of this review?**

This review summarizes the evidence from six randomized controlled trials that judged the effectiveness of systematic review summaries on policymakers' decision making, or the most effective ways to present evidence summaries to increase policymakers' use of the evidence.

**What are the main findings of this review?**

This review included six randomized controlled studies. A randomized controlled study is one in which the participants are divided randomly (by chance) into separate groups to compare different treatments or other interventions. This method of dividing people into groups means that the groups will be similar and that the effects of the treatments they receive will be compared more fairly. At the time the study is done, it is not known which treatment is the better one.

The researchers who did these studies invited people from Europe, North America, South America, Africa, and Asia to take part in them. Two studies looked at “policy briefs,” one study looked at an “evidence summary,” two looked at a “summary of findings table,” and one compared a “summary of findings table” to an evidence summary.

None of these studies looked at how policymakers directly used evidence from systematic reviews in their decision making, but two studies found that there was little to no difference in how they used the summaries. The studies relied on reports from decision makers. These studies included questions such as, “Is this summary easy to understand?”

Some of the studies looked at users' knowledge, understanding, beliefs, or how credible (trustworthy) they believed the summaries to be. There was little to no difference in the studies that looked at these outcomes. Study participants rated the graded entry format higher for usability than the full systematic review. The graded entry format allows the reader to select how much information they want to read.. The study participants felt that all evidence summary formats were easier to understand than full systematic reviews.

**What do the findings of this review mean?**

Our review suggests that evidence summaries help policymakers to better understand the findings presented in systematic reviews. In short, evidence summaries should be developed to make it easier for policymakers to understand the evidence presented in systematic reviews. However, right now there is very little evidence on the best way to present systematic review evidence to policymakers.

**How up to date is this review?**

The authors of this review searched for studies through June 2016.

**Executive summary/Abstract:**

**Background**

Systematic reviews are important for decision makers. They offer many potential benefits but are often written in technical language, are too long, and do not contain contextual details which makes them hard to use for decision‐making. Strategies to promote the use of evidence to decision makers are required, and evidence summaries have been suggested as a facilitator. Evidence summaries include policy briefs, briefing papers, briefing notes, evidence briefs, abstracts, summary of findings tables, and plain language summaries. There are many organizations developing and disseminating systematic review evidence summaries for different populations or subsets of decision makers. However, evidence on the usefulness and effectiveness of systematic review summaries is lacking. We present an overview of the available evidence on systematic review evidence summaries.

**Objectives**

This systematic review aimed to 1) assess the effectiveness of evidence summaries on policy‐makers' use of the evidence and 2) identify the most effective summary components for increasing policy‐makers' use of the evidence.

**Search methods**

We searched several online databases (Medline, EMBASE, CINAHL, Cochrane Central Register of Controlled Trials, Global Health Library, Popline, Africa‐wide, Public Affairs Information Services, Worldwide Political Science Abstracts, Web of Science, and DfiD), websites of research groups and organizations which produce evidence summaries, and reference lists of included summaries and related systematic reviews. These databases were searched in March‐April, 2016.

**Selection criteria**

Eligible studies included randomised controlled trials (RCTs), non‐randomised controlled trials (NRCTs), controlled before‐after (CBA) studies, and interrupted time series (ITS) studies. We included studies of policymakers at all levels as well as health system managers. We included studies examining any type of “evidence summary”, “policy brief”, or other product derived from systematic reviews that presented evidence in a summarized form. These interventions could be compared to active comparators (e.g. other summary formats) or no intervention.

The primary outcomes were: 1) use of systematic review summaries decision‐making (e.g. self‐reported use of the evidence in policy‐making, decision‐making) and 2) policymaker understanding, knowledge, and/or beliefs (e.g. changes in knowledge scores about the topic included in the summary). We also assessed perceived relevance, credibility, usefulness, understandability, and desirability (e.g. format) of the summaries.

**Results**

Our database search combined with our grey literature search yielded 10,113 references after removal of duplicates. From these, 54 were reviewed in full text and we included 6 studies (reported in 7 papers, 1661 participants) as well as protocols from 2 ongoing studies. Two studies assessed the use of evidence summaries in decision‐making and found little to no difference in effect. There was also little to no difference in effect for knowledge, understanding or beliefs (4 studies) and perceived usefulness or usability (3 studies). Summary of Findings tables and graded entry summaries were perceived as slightly easier to understand compared to complete systematic reviews. Two studies assessed formatting changes and found that for Summary of Findings tables, certain elements, such as reporting study event rates and absolute differences were preferred as well as avoiding the use of footnotes. No studies assessed adverse effects. The risks of bias in these studies were mainly assessed as unclear or low however, two studies were assessed as high risk of bias for incomplete outcome data due to very high rates of attrition.

**Authors' conclusions**

Evidence summaries may be easier to understand than complete systematic reviews. However, their ability to increase the use of systematic review evidence in policymaking is unclear.

## Background

Policy makers are increasingly utilizing systematic reviews for decision‐making (Lavis et al., 2006; Moat et al.; Petticrew et al., 2004; Welch et al., 2012). The shift from single studies has occurred because systematic reviews offer additional benefits to policymakers, such as having lower risk of bias than other studies, and offering more confidence in results than single studies (Lavis et al., 2006a). However, health policies are often made without the use of research evidence (Oxman et al., 2009). Barriers to the use of research, specifically systematic reviews, in policymaking have been identified (Oliver et al., 2014). Systematic reviews are often written using technical language, lack important contextual information and can be quite long. Because of this, research groups and organizations have begun creating summaries of the evidence (Moat et al., 2013; Rosenbaum et al., 2011). Strategies to promote the use of research evidence to policy‐makers are required, and evidence summaries have been suggested as a facilitator to evidence‐informed decision‐making (Bunn & Sworn, 2011).

### The problem, condition or issue

There are several organizations that develop and disseminate evidence summaries for different populations or subsets of decision makers. For example, within the Cochrane Collaboration, the Evidence Aid Project was developed in response to the 2004 Indian Ocean Tsunami as a means of providing decision makers and health practitioners ‘on the ground’ with summaries of the best available evidence needed to respond to emergencies and natural disasters (Kayabu et al., 2013).

SUPPORT Summaries were developed for policy‐makers in low‐ and middle‐income countries (LMICs) making decisions about maternal and child health programs and interventions (www.support‐collaboration.org). Health Systems Evidence provides a one‐stop shop for systematic reviews related to health systems including policy briefs for policy‐makers and other stakeholders (www.healthsystemsevidence.org/). Other examples include Cochrane Summaries (http://www.cochrane.org/evidence), Communicate to vaccinate (COMMVAC (http://www.commvac.com), Rx for change (www.cadth.ca/resources/rx‐for‐change), and Harvesting Evidence (http://www.harvesting‐evidence.org). A document analysis conducted by Adam et al. identified 16 organizations involved in the production of summaries for policymakers in LMICs (Adam et al., 2014).

A needs assessment conducted by Evidence Aid found that while complete systematic reviews were perceived to be useful for workers ‘on the ground’ (i.e. Non‐Governmental Organizations (NGOs), health care providers), summaries containing contextual information were considered helpful for decision‐making about the applicability of the findings to their local setting (Kayabu et al., 2013).

### The intervention

Evidence summaries of systematic reviews are identified using many different terms including evidence summaries, policy briefs, briefing papers, briefing notes, evidence briefs, abstracts, summary of findings, and plain language summaries (Adam et al., 2014). They are intended to assist decision makers in understanding the evidence and encourage its use in their decision‐making. These user‐friendly formats highlight the policy‐relevant information and allow policymakers to quickly scan the document for relevance (Lavis et al., 2005a; Lavis et al., 2006a). The various products have some differences. For example, abstracts, evidence summaries, and summary of findings tables usually summarize evidence from a single systematic review while policy briefs often utilize evidence from one or more systematic reviews and may use additional sources to provide contextual or economic information (Adam et al., 2014).

### How the intervention might work

Systematic review summaries consist of summarized evidence from systematic reviews intended to assist policy‐makers in understanding the systematic review evidence and using it in their decision‐making. These interventions may include structured summaries (e.g. SUPPORT summaries, Evidence Aid), policy briefs which are based on systematic reviews (e.g. Health Systems Evidence), and plain language summaries, structured abstracts, and Summary of Findings tables (e.g. Cochrane reviews). These may be provided in print or web‐based formats and are aimed at policy‐makers, and other decision makers making decisions about health. The summaries may include information about the context in which the studies were conducted, the applicability of the results (e.g. SUPPORT Summaries comment on the relevance of the findings for disadvantaged communities), as well as the findings, methods and conclusions.

Evidence suggests that policy makers are more likely to use systematic reviews when the evidence is provided in a timely manner, and aligns with interests, values, and political goals of policymakers (Lavis et al., 2005a; Lavis et al., 2005b). Evidence summaries may increase the use of systematic review evidence by policymakers because they fulfil these by: 1) providing “user friendly” and plain language summaries of the evidence, 2) providing evidence “at‐a‐glance” with links to the complete systematic reviews, and 3) focusing on policy‐relevant topics (Adam et al., 2014; Lavis et al., 2009). In addition, they may improve access to systematic reviews, because most organizations make summaries available freely through online databases and repositories (Adam et al., 2014).

### Why it is important to do the review

Interest in the production and use of systematic review summaries is increasing, as evidenced by the growing number of organizations developing and disseminating them (Adam et al., 2014). However, evidence on the usefulness and effectiveness of systematic review derivatives is lacking. Previously conducted systematic reviews have looked at interventions to increase the use of systematic reviews among decision makers. However, these have focused on the use of complete systematic reviews in decision‐making and none focused specifically on derivatives of systematic reviews. For example, one systematic review examined the effectiveness of interventions for improving the use of systematic reviews in decision‐making by health system managers, policymakers, and clinicians (Murthy et al., 2012). This review included eight studies and the authors concluded that information provided as a single, clear message may improve evidence‐based practice but increasing awareness and knowledge of systematic review evidence might require a multi‐faceted intervention. Similarly, another systematic review assessed interventions encouraging the use of systematic reviews by health policymakers and managers (Perrier et al., 2011). Four studies were included and the authors concluded that future research should identify how systematic reviews are accessed and the formats used to present the information. A systematic review by Wallace et al. found that a description of benefits as well as harms and costs, and using a graded entry approach (in which evidence is available as a 1 page summary, 3 page summary, or 25 page full report) facilitated systematic review use by policymakers (Wallace et al., 2012). Similarly, a systematic review by Oliver et al. also assessed barriers and facilitators to the use of research by policymakers; they found that access to high quality, relevant research as well as collaboration between researchers and policymakers were the most important factors for increasing research use (Oliver et al., 2014). In addition, we focused on studies of evidence summaries for health policy‐makers and health system managers making decisions on behalf of a large jurisdiction or organization but did not include studies related to decision‐making for an individual person or patient.

## Objectives

The objectives of this review were to:

1) assess the effectiveness of evidence summaries on policy‐makers' use of the evidence and;
2) identify the most effective summary components for increasing policy‐makers' use of the evidence.

## Methods

The protocol for this review was published in the Campbell Library on 3 August 2017.

### Criteria for considering studies for this review

#### Types of studies

Eligible studies included randomised controlled trials (RCTs), non‐randomised controlled trials (NRCTs), controlled before‐after (CBA) studies, and interrupted time series (ITS) studies.

#### Types of participants

We included studies whose participants were health policymakers at all levels. We defined policymakers as health ministers and their political staff, civil servants, and health system managers, and health‐system stakeholders as civil society groups, patient groups, professional associations, non‐governmental organizations, donors, international agencies (Lavis et al., 2004). We included populations involved in the development of clinical practice guidelines. To be included, the population had to be responsible for decision‐making on behalf of a jurisdiction or organization and we did not include studies related to decision‐making for an individual person or patient (Lavis et al., 2004; Perrier et al., 2011). For the purposes of this review, we defined ‘health policy‐makers’ as those responsible for making decisions about healthcare policies and programs which are those intended to restore or maintain physical, mental, or emotional wellbeing (WHO, 2017). We included studies in which there were mixed participants as long as some participants are directly involved in health policy‐making.

#### Types of interventions

We included studies of interventions examining any type of “friendly front end”, “evidence summary”, or “policy brief” or other product derived from systematic reviews or guidelines based on systematic reviews that presents evidence in a summarized form to policy‐makers and health system managers. Interventions had to include a summary of a systematic review and be actively “pushed” to target users. This means that the summary had to be disseminated or shown to the study participants via email, mail or other means. We included any comparisons including active comparators (e.g. other summary formats) or no intervention.

#### Types of outcome measures

##### Primary Outcomes

1. Use of systematic review derivative product in decision‐making (e.g. self‐reported use of the evidence in policy‐making, decision‐making as well as self‐reported access of research, appraisal of research, or commissioning of further research within the decision‐making process (Redman et al., 2015). We defined “use” as instrumental, conceptual, or symbolic use of research in decision‐making. Instrumental use of is the direct use of research, conceptual use includes using research to gain an understanding of a problem or intervention, and symbolic use is the use of research to confirm a policy or program already implemented (Amara, et al., 2004).
2. Understanding, knowledge, and/or beliefs (e.g. changes in knowledge scores about the topic included in the summary) as reported by the authors of the included studies (Amara et al., 2004).

##### Secondary Outcomes

- Perceived relevance of systematic review summaries
- Perceived credibility of the summaries
- Perceived usefulness and usability of systematic review summaries
  ○ Perceptions and attitudes regarding the specific components of the summaries and their usefulness
- Understandability of summaries
- Desirability of summaries (e.g. layout, selection of images, etc.) (Rosenbaum et al., 2011)

Some studies may use different terms to describe these outcomes, therefore, our team assessed each outcome and categorized them according to the above list. Studies were not excluded on the basis of outcomes. Outcome measures may include Likert scales to assess understandability, credibility, likelihood to use a summary in decision‐making, or other outcomes; knowledge scores or responses to questions regarding summary content; and preferred format styles.

Two reviewers independently screened titles and abstracts to identify relevant studies meeting the pre‐specified inclusion criteria. The full text of each potentially included study was then screened independently by two authors.

### Search methods for identification of studies

#### Electronic searches

Information Specialists (APA, HC) developed and translated the search strategy using the PRESS Guideline (Sampson et al., 2008). We expected that the indexing for eligible studies would be poor. Therefore, our search strategy was intentionally broad and we were prepared to retrieve a high number of citations for a low yield of included studies.

We used the search strategy developed by Perrier et al. and Murthy et al. for their systematic reviews of interventions to encourage the use of systematic reviews by health managers and policymakers to inform our search (Murthy et al., 2012; Perrier et al., 2011). This search included the following databases: Medline, EMBASE, CINAHL, Cochrane Central Register of Controlled Trials. We expanded the Perrier search by including additional databases, as suggested by John Eyres, of the International Initiative for Impact Evaluation (3ie) and the Campbell International Development Review Group. These included Global Health Library (from WHO), Popline, Africa‐wide, Public Affairs Information Service, Worldwide Political Science Abstracts, Web of Science, and DfiD (Research for Development Database). The search strategies were translated using each database platform's command language and appropriate search fields. Both controlled vocabulary terms and text‐words were used for the search concepts of policymaking, evidence synthesis, systematic reviews, knowledge translation, and dissemination. No date restrictions were used. The complete MEDLINE search strategy is available in online supplement 1. All databases were searched in March‐April, 2016.

#### Searching other resources

We identified and searched websites of research groups and organizations which produce evidence summaries building on the list of organizations identified by Adam et al. (Adam et al., 2014). We searched for unpublished studies evaluating the effectiveness of the systematic review derivatives in increasing policymakers' understanding (e.g. Health Systems Evidence, the Canadian Agency For Drugs And Technologies In Health, SUPPORT Summaries). A complete list of grey literature sources is provided in online supplement 2 and these sources were searched in June, 2016.

**Figure 1:**
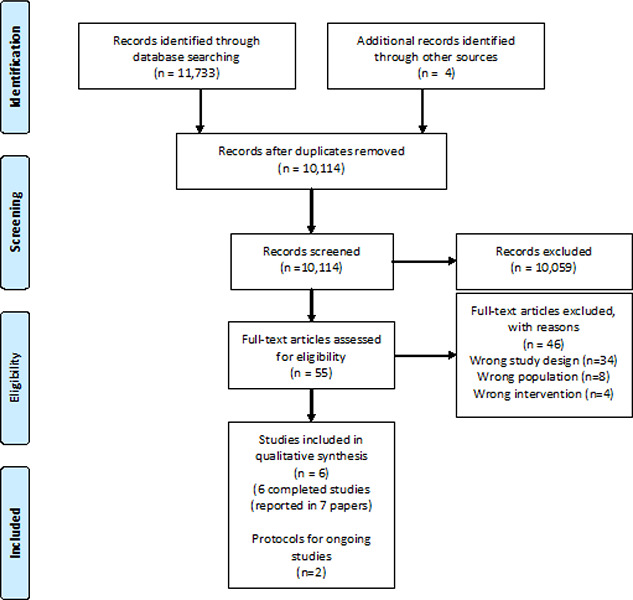
PRISMA Flow Diagram

We also checked the reference lists of included studies and related systematic reviews to identify additional studies. We contacted researchers to identify ongoing and completed work. The results of the search are reported in Figure 1.

### Data collection and analysis

#### Selection of studies

References identified through database and grey literature searching were screened independently, in duplicate by two members of the research team using Covidence software.

#### Data extraction and management

The data extraction form was pre‐tested, and included factors related to the population, intervention, comparison, and outcomes. Data extraction was completed by two authors independently using a structured Excel sheet. Disagreements were resolved by discussion and with a third member of the research team when necessary.

Data were extracted for the following:

- Country
- Setting
- Study design
- Participants
  ○ Type of policy or decision makers
  ○ Country
  ○ Age
  ○ Gender and/or sex
- Intervention
  ○ Type of evidence summary
  ○ Format of evidence summary
  ○ Description of evidence summary components (e.g. descriptions of easy‐to‐skim formatting, graded entry, use of tables and/or figures)(Moat et al.)
  ○ Mode of delivery
  ○ Topic of evidence summary
  ○ Recommendation of evidence summary
- Outcomes
  ○ Policy‐ and/or decision makers' self‐reported use of summaries in decision‐making
  ○ Policy‐ and/or decision makers' knowledge of the summary content and the measurement used
  ○ Policy‐ and/or decision makers' understanding and measurement used
  ○ Perceived relevance of the summaries and measurement used
  ○ Perceived credibility of the summaries and measurement used
  ○ Perceived usefulness and usability of the summaries and measurement used
  ○ Perceived understandability of the summaries and measurement used
  ○ Perceived desirability of the summaries and measurement used
- Process Indicators
  ○ How the systematic review was selected for summary (e.g. based on topic, quality criteria)
  ○ How the evidence summary was developed (e.g. iterative process)
  ○ Involvement of stakeholders in evidence summary development – which stakeholders, description of involvement

#### Assessment of risk of bias in included studies

The methodological quality was assessed using the risk of bias tool from the Cochrane Handbook for randomized trials. If we had identified eligible ITS, CBA, or NRS we planned to use the Effective Practice and Organization of Care (EPOC) Review Group criteria for ITS and CBA studies (Ballini et al., 2011; Higgins & Green, 2011) and Risk Of Bias In Non‐randomized Studies ‐ of Interventions (ROBINS‐I) (Sterne et al., 2014; M. G. Wilson et al., 2015).

We used the Grading of Recommendations Assessment, Development and Evaluation (GRADE) approach to assess the quality of evidence for the outcomes reported in this review (Guyatt et al., 2011).

#### Dealing with missing data

We attempted to contact the authors of studies with missing data and the authors of ongoing studies.

#### Assessment of heterogeneity

Our included studies assessed interventions and reported outcomes that were too different to pool; therefore, meta‐analysis was not possible. If it had been, we planned to explore heterogeneity using forest plots and the I^2^ statistic according to guidance of the Cochrane Handbook for Systematic Reviews of Interventions (Higgins et al.). We were also thus unable to conduct planned meta‐regression to assess the role of mediating factors, such as: target audience of summary (e.g. focused on specific local context, generic summary); type of decision maker (e.g. federal policy‐maker versus hospital administrator); and components of friendly front end (e.g. bulleted list, text, summary of findings table, causal chain).

#### Assessment of reporting biases

We did not assess publication bias using funnel plots because only six studies were included.

#### Data synthesis

We planned to synthesize the results using meta‐analysis, if possible but, as stated in the protocol, we planned to present a narrative summary if the results could not be pooled. The interventions assessed in the studies we have included were too different with respect to their methods, populations, and outcomes to meaningfully pool results across studies.

#### Subgroup analysis and investigation of heterogeneity

Meta‐analysis was not possible therefore we were unable to conduct subgroup analyses or assess heterogeneity.

#### Sensitivity analysis

We planned to assess the impact of including studies assessed as high risk of bias or studies in which there were unit of analysis errors that could not be reanalysed but meta‐analysis was not possible.

## Results

### Description of studies

#### Results of the search

The search strategy yielded 11,733 references (10,113 after removal of duplicates). Figure 1 depicts the results of the search and screening. During the title and abstract screening process we excluded 10,059 references for failing to meet one or more of our inclusion criteria. The remaining 50 references were reviewed as full‐text plus three additional references identified through reference‐list checking and one additional reference identified through grey literature searching. We excluded 45 studies that did not meet our eligibility criteria (see online supplement 1).

#### Included studies

We included six completed RCTs reported in seven articles in this review (Beynon et al., 2012; Brownson et al., 2011; Carrasco‐Labra et al., 2016; Dobbins et al., 2009; Masset et al., 2013; Opiyo et al., 2013; Vandvik et al., 2012). The characteristics of the included studies are summarized in table 1.

**Table 1:** Characteristics of included studies

**Study ID**	**Methods**	**Study dates**	**Funding**	**Participants**	**Intervention description**	**Outcomes**
Brownson 2011 (Brownson et al., 2011)	RCT	Data collected between Feb. 2, 2009 and Dec. 31, 2009.	National Cancer Institute at the National Institutes of Health	Legislative staff members (e.g. committee staff), state legislators, and executive branch administrators (e.g. division directors, program heads) (n=291 participants)	Each participant was e‐mailed 1 of the 4 briefs. 4 different policy briefs on mammography screening to reduce breast cancer mortality: data‐focused brief with state‐level data, data‐focused brief with local‐level data, story‐focused brief with state‐level data, and story‐focused brief with local‐level data.	Self‐reported understandability (using 3 measures assessing whether the information was presented clearly, in an attractive way and held the reader's attention) and credibility (2 measures that assessed whether the information in the brief was believable and accurate).
Carrasco‐Labra 2016 (Carrasco‐Labra et al., 2016)	RCT	Dates not provided	Cochrane Methods Innovation Fun, GRADE Center at McMaster University	Health care professionals, guideline developers and researchers that use and/or develop systematic reviews (n=290 participants)	An alternate Summary of Findings table was compared against the current format: alternate format provides options to display the same data in a different way or to provide supplementary data to the current format.	Self‐reported understanding assessed with 7 multiple‐choice questions (5 response options). Self‐reported accessibility of information assessed with 3 self‐reported domains (how easy it is to find critical information, how easy it is to understand the information, whether the information is presented in a useful way for decision‐making. Satisfaction measured by asking which about satisfaction with the different formatting elements. Preference assessed using a 7‐point Likert scale for the 2 tables.
Dobbins 2009 (Dobbins et al., 2009)	RCT	Baseline assessment Sept. – Nov. 2004, intervention in 2005, final assessment between Jan. and Mar. 2006	Canadian Institutes of Health Research	Front line staff, managers, directors, coordinators and others from public health departments in Canada (those directly responsible for making program decisions related to healthy body weight promotion in children) (n=108 participants – representing health departments)	1^st^ group (control): access to health‐evidence.ca and received an email about access to this resource 2^nd^ group: received tailored, targeted messages ‐ 7 emails with titles of 7 high‐quality SRs related to health body weight promotion in children and links to full text, abstract, and summary, plus access to health‐evidence.ca 3^rd^ group: same intervention as the 2nd group plus access to a full‐time knowledge broker who was available to provide relevant research to provided the decision makers in a way that was useful, helped them to develop skills for evidence‐informed decision‐making, and translating the evidence.	Self‐reported global evidence‐informed decision‐making (participants were asked to report the extent to which research evidence was considered in a recent program planning decision within the previous 12 months) related to healthy body weight and promotion and public health policies and programs measured by the sum of actual strategies, policies, and/or interventions for healthy body weight promotion in children being implemented by the department.
Masset 2013 (Beynon et al., 2012; Masset et al., 2013)	RCT	Summer 2011	International Initiative for Impact Evaluation (3ie)	Individuals who normally read policy briefs related to international development ‐ e.g. employed in academia, NGOs and international aid organizations ‐ some self‐reported influence on policy decisions and therefore considered policymakers (n=807 participants)	3 versions of a policy brief summarising the results of a SR: one group received a link to a standard policy brief via email, 2^nd^ group received a link to a policy brief with director's commentary via email 3^rd^ group received a link to the policy brief with unnamed research fellow's commentary via email	Beliefs about the effectiveness of and strength of the evidence for the interventions included in the briefs.
Opiyo 2013 (Opiyo et al., 2013)	RCT	June 2010	Wellcome Trust Strategic Award	Panel of healthcare professionals with roles in neonatal and pediatric policy and care in Kenya (n=77 participants)	3 intervention packages: pack A contained a systematic review alone, pack B included systematic reviews with summary of findings tables, and pack C received an evidence summary with a graded entry format Participants attended a workshop at baseline and then completed a follow up face‐to‐face interview	Self‐reportedunderstanding of the summary content measured by the proportion of correct responses to clinical questions relevant to the effects of the intervention. Value and accessibility (usefulness and usability) of the evidence was assessed using a 3 or 5‐point scale.
Vandvik 2012 (Vandvik et al., 2012)	RCT	Dates not provided	No funding source provided	All panelists for the Antithrombotic therapy and prevention of thrombosis, American College of Chest Physicians (n=88 participants)	2 formats of the evidence profile that differed by 4 features were emailed to participants: placement of additional information, placement of overall quality of evidence, study event rates, absolute risk differences Each group received 1 of 4 emails with similar text but different links allowing download of the evidence profile.	User preferences for specific formatting options and the overall format of the table were assessed using a 7‐point Likert scale. Comprehension of key findings was assessed with multiple choice questions. Accessibility of the information for quality of evidence and relative and absolute effects was assessed using 3 domains: easy to find, easy to understand, and helpful in making recommendation using a 7‐point scale. Time needed to comprehend information about quality assessment and key findings was assessed by asking participants to record the time before and after answering questions testing comprehension.

The completed studies recruited participants from Canada (n=1), Kenya (n=1), the US (n=1), internationally without specifying countries (46% from high‐income countries) (n=1), and in countries in Europe, North America, South America, Africa, and Asia (n=1) (Beynon et al., 2012; Brownson et al., 2011; Carrasco‐Labra et al., 2016; Dobbins et al., 2009; Masset et al., 2013; Opiyo et al., 2013). One study did not report participants' country (Vandvik et al., 2012). Additionally, we identified two protocols for eligible studies: one RCT (Wilson et al., 2011) and one CBA (Wilson et al., 2015). These ongoing studies will be conducted in Canada (n=1) and the United Kingdom (n=1) (Wilson et al., 2011; Wilson et al., 2015). The details of these studies are presented in table 2.

**Table 2:** Characteristics of ongoing studies

**Study ID**	**Methods**	**Participants**	**Intervention description**	**Outcomes**
Wilson 2011 (Wilson et al., 2011)	RCT	Decision‐makers, (programs, services, advocacy) from community‐based HIV/AIDS organizations in Canada affiliated with the Canadian AIDS Society and from relevant provincial HIV/AIDS networks	At baseline, all participants will receive the ‘evidence service (includesa listing of relevant systematic reviews, links to PubMed records, and worksheets to help find and use research evidence). During the intervention, one group will receive the ‘full‐serve’ version of SHARE (‘Synthesized HIV/AIDS Research Evidence’) which includes access to a database of HIV systematic reviews, emailed updates, access to user‐friendly summaries, links to scientific abstracts, peer relevance assessments (indicating how useful the information is), as well as an interface for comments in the records, plus links to the full‐text, and access to worksheets to help find and use evidence. The control group will continue to receive the ‘self‐serve’ evidence service. During the final two‐month period, both groups will receive the ‘full‐serve’ version of SHARE.	The primary outcome measure will be the mean number of logins/month/organization. The secondary outcome will be intention to use research evidence (measured with a survey administered to one key decision maker from each organization).
Wilson 2015 (P. M. Wilson et al., 2015)	CBA	Clinical Commissioning Groups: Governing body and executive members, clinical leads and any other individuals deemed as being involved in commissioning decision‐making processes	Three arms: 1) consulting plus responsive push of tailored evidence (access to an evidence briefing service provided by the Centre for Reviews and Dissemination (CRD) plus advice and support via phone, email, face‐to‐face; monthly check in to discuss further evidence needs; issues around use of evidence; alert team to new SRs and other synthesized evidence relevant to priorities); 2) consulting plus an unsolicited push of non‐tailored evidence (access to intervention 1 without tailored evidence briefings and instead just evidence briefings without contextual information); or 3) ‘standard’ service (CRD will disseminate evidence briefings generated in intervention 1 and any other non‐tailored briefings produced by CRD over the intervention period).	Primary outcome: change at 12 months from baseline of a CCGs ability to acquire, assess, adapt and apply research evidence to support decision‐making. Secondary outcomes will measure individuals' intentions to use research evidence in decision‐making.

##### Description of the interventions

Details of the different evidence summary formats are reported in table 3. Briefly, two studies assessed policy briefs (Brownson et al., 2011; Masset et al., 2013), one assessed an “evidence summary”(Dobbins et al., 2009), two assessed different formats of summary of findings tables, which are distinct table formats presenting the main findings of the review (absolute and relative effects for each important outcome) and quality of the evidence (Carrasco‐Labra et al., 2016; Vandvik et al., 2012), and one compared a Summary of Findings table alone to a summary of findings table as part of a “graded entry” evidence summary (a short one page summary, then a narrative report, followed by access to the complete systematic review) (Opiyo et al., 2013). Two studies assessed evidence summaries which included recommendations for programs or policies, (Brownson et al., 2011; Dobbins et al., 2009) while the others did not specify whether recommendations were provided within the summary (Masset et al., 2013; Opiyo et al., 2013; Vandvik et al., 2012).

**Table 3:** Evidence summary formats and results

**Study**	**Type of evidence summary**	**Format of summary**	**Method of delivery**	**Components**	**Outcomes**
Brownson 2011 (Brownson et al., 2011)	Policy brief	Printed leaflet/booklet, pdf version for those who prefer online	Mailed, follow up telephone call, emailed if preferred	Front cover varied according to story vs data driven, colour printed (included data or story), 3rd and 4th pages the same across all 4 briefs, data driven briefs contained 2 statements with percentages related to mammography screening, story driven had 2 personal stories related to mammography, all briefs had data about uninsured women, women not up to date on mammograms, breast cancer mortality compared to other causes, benefits of mammograms, and recommendations	The briefs were considered understandable and credible (mean ratings ranged from 4.3 to 4.5 on 5 point Likert scale). Likelihood of using the brief was different by study condition for staff members (P = .041) and legislators (P = .018). Staff members found the story‐focused brief with state‐level data the most useful. Legislators found the data‐focused brief with state‐level data most useful.
Carrasco‐Labra 2016 (Carrasco‐Labra et al., 2016)	Summary of Findings table	Table	Emailed link to online survey	The new format of Summary of Findings table moved the number of participants and studies to the outcomes column, quality of evidence was presented with the main reasons for downgrading, “footnotes” was changed to “explanations”, baseline risk and corresponding risk were expressed as percentages, column presenting absolute risk reduction (risk difference) or mean difference, no comments column, Addition of “what happens” column, no description of the GRADE evidence definitions.	Participants with the new Summary of Findings table format had higher proportion of correct answers for almost all questions. The new format was more accessible, measure by reports of it being easier to understand information about the effects (MD 0.4, SE 0.19); and displayed results in a way that was more helpful for decision‐making (MD 0.5 SE 0.18), Overall, participants preferred the new format (MD 2.8, SD 1.6).
Dobbins 2009 (Dobbins et al., 2009)	Evidence Summaries	text	Targeted, tailored emails	Short summary including key findings and recommendations	The post intervention change in Global Evidence‐Informed Decision‐making was 0.74 (95% CI 0.26‐1.22) for the group receiving only access to healthevidence.org; ‐0.42 (‐1.10, 0.26) for the group receiving tailored, targeted emails; and ‐0.09 (‐0.78, 0.60) for the knowledge broker group. The changes in health policies and programs (HPP) after the intervention were ‐0.28 (‐1.20, 0.65) for the group receiving only access to the healthevidence.org website; 1.67 (0.37, 2.97) for the group receiving tailored, targeted messages; and ‐0.19 (‐1.50, 1.12) for the group with access to a knowledge brokers. The tailored, targeted messages are more effective than the knowledge broker intervention or access to www.health‐evidence.ca in organizations with a culture that highly values research.
Masset 2013 (Beynon et al., 2012; Masset et al., 2013)	Policy Brief	text, colour leaflet	Email	Introduction to the problem, description of methodology, conclusions and policy implications, 2 versions had expert commentary	Respondents with stronger beliefs about the agricultural interventions at baseline rated the policy brief more favourably. The policy brief was less effective in changing respondents' ratings of the strength of the evidence and effectiveness of the intervention.
Opiyo 2013 (Opiyo et al., 2013)	Summary of Findings table, graded entry summary of evidence	text, tables	Email	Summary of Findings table Graded entry format included a summary and interpretation of main findings and conclusions, a contextually framed narrative report, and Summary of Findings table	No differences between groups in the odds of correct responses to key clinical questions. Both packs B and C improved understanding. Pack C compared to pack A was associated with a significantly higher mean ‘value and accessibility’ score. Pack C compared to pack A, was associated with a 1.5 higher odds of judgments about the quality of evidence being clear and accessible. More than half of participants preferred narrative report formats to the full version of the SR (53% versus 25%). A higher respondent percentage (60%) found SRs to be more difficult to read compared to narrative reports, but some (17%) said that SRs were easy to read. About half of the participants (51%) found SRs to be easier to read compared to summary‐of‐findings tables (26%).
Vandvik 2012 (Vandvik et al., 2012)	Summary of Findings table	table	Email	Tables presented outcomes, number of participants, summary of findings, and quality assessment using GRADE	Participants liked presentation of study event rates over no study event rates, absolute risk differences over absolute risks, and additional information in table cells over footnotes. Panelists presented with time frame information in the tables, and not only in footnotes, were more likely to properly answer questions regarding time frame and those presented with risk differences and not absolute risks were more likely to rightly interpret confidence intervals for absolute effects. Information was considered easy to find and to comprehend, and also helpful in making recommendations regardless of table format.

Carrasco‐Labra et al. compared a standard format summary of findings table to a new format that presented some of the data in a different way as well as provided supplementary data (Carrasco‐Labra et al., 2016). All the other included studies tested evidence summary formats using multiple arms. Brownson et al. compared four versions of a policy brief: a state‐level data‐focused brief, a local‐level data‐focused brief, a story‐focused brief with state‐level data, and a story‐focused brief with local‐level data (Brownson et al., 2011).

Dobbins et al. had three groups. The first had access to the online database, the second received targeted, tailored messages in addition to access to an online database, and the third group received the same intervention as the second group plus access to a full‐time knowledge broker (Dobbins et al., 2009).

Masset et al., and the companion paper by Beynon et al., assessed three versions of a policy brief. The first was the standard policy brief, the second was the same policy brief with an additional commentary by a sector expert (the Director of the institution who conducted the review), and the third was the same except the commentary was attributed to an unnamed research fellow (Beynon et al., 2012; Masset et al., 2013).

The study by Opiyo et al. compared a systematic review alone to a systematic review with a summary of findings table and a ‘graded‐entry format’ which includes a short one page summary, a contextually‐framed narrative report including an interpretation of the main findings and conclusions (with a summary of findings table), followed by access to the full systematic review (Opiyo et al., 2013).

Finally, the study by Vandvik et al. compared two versions of summary of findings tables with or without four formatting modifications (the placement of additional information, the placement of the overall rating for quality of evidence, the study event rates, and the absolute risk differences) (Vandvik et al., 2012). These were labelled as Table A and Table B. Table A contained additional information within the table, an overall assessment of the quality of evidence in a ‘quality assessment’ heading, and reported the absolute risk differences but not the study event rates. Table B presented the additional information in footnotes, included the overall quality of the evidence under a “summary of findings' heading, reported study event rates, but did not report absolute risk differences.

#### Excluded studies

After title and abstract screening, 10,059 references were excluded because they did not meet our eligibility criteria. After reviewing 55 full text references an additional 46 studies were excluded. The details of these exclusions are included in online supplement 3.

### Risk of bias in included studies

**Figure 2:**
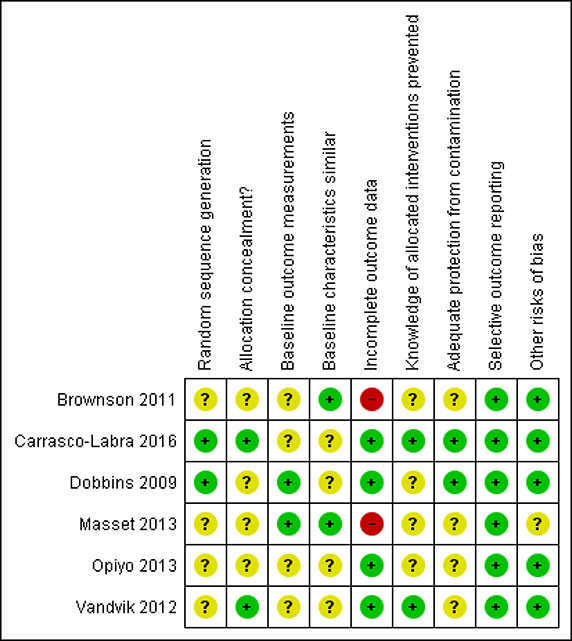
Risk of bias

The summary of the Risk of Bias assessments is presented in Figure 2 and details are provided in online supplement 4.

#### Random sequence generation

Two studies were assessed as low risk of bias for random sequence generation (Carrasco‐Labra et al., 2016; Dobbins et al.) and the others were assessed as unclear (Brownson et al., 2011; Masset et al., 2013; Opiyo et al., 2013; Vandvik et al., 2012).

#### Allocation concealment

For allocation concealment, four studies were assessed as unclear (Beynon et al., 2012; Brownson et al., 2011; Dobbins et al., 2009; Masset et al., 2013; Opiyo et al., 2013) and two studies assessed as low risk of bias (Carrasco‐Labra et al., 2016; Vandvik et al., 2012).

#### Baseline outcome measures and characteristics

Baseline outcome measurements were similar and therefore low risk of bias in two studies (Beynon et al., 2012; Dobbins et al., 2009; Masset et al., 2013) and unclear in four (Brownson et al., 2011; Carrasco‐Labra et al., 2016; Opiyo et al., 2013; Vandvik et al., 2012). Baseline characteristics were also similar in two studies (Beynon et al., 2012; Brownson et al., 2011; Masset et al., 2013) and unclear in the others (Carrasco‐Labra et al., 2016; Dobbins et al., 2009; Opiyo et al., 2013; Vandvik et al., 2012).

#### Incomplete outcome data

Incomplete outcome data was assessed as low risk of bias for four studies (Carrasco‐Labra et al., 2016; Dobbins et al., 2009; Opiyo et al., 2013; Vandvik et al., 2012) but high for two studies (Beynon et al., 2012; Brownson et al., 2011; Masset et al., 2013). These two studies had very high rates of attrition; Brownson et al. had an overall response rate of 35% and the Masset study had 50% attrition between baseline and first follow‐up (Brownson et al., 2011).

#### Knowledge of allocated interventions

Knowledge of allocated interventions was assessed as unclear for four of the studies (Beynon et al., 2012; Brownson et al., 2011; Dobbins et al., 2009; Masset et al., 2013; Opiyo et al., 2013). One study reported that panelists, data collection, and data analysis were blinded (Vandvik et al., 2012) and one reported that allocation was done in real‐time when the survey was completed and these were therefore assessed as low risk of bias (Carrasco‐Labra et al., 2016).

#### Protection from contamination

Adequate protection from contamination was assessed as unclear for four studies. The Dobbins study included public health departments from across Canada and therefore little risk of contamination was expected (Dobbins et al., 2009) and Carrasco‐Labra et al. reported that allocation was done in real‐time when completing the survey leaving little risk of contamination (Carrasco‐Labra et al., 2016).

#### Selective outcome reporting

All studies were assessed as low risk of bias for selective outcome reporting.

#### GRADE

Most outcomes were assessed as moderate certainty of evidence using GRADE (Guyatt et al., 2011). Reasons for downgrading the evidence were due to unclear risk of bias. ‘Perceived credibility’ was assessed as low certainty of evidence due to unclear risk of bias and only one eligible study for this outcome.. The assessments are included in table 4.

**Table 4:** Summary of findings

**Evidence summaries to increase policymakers' use of systematic review evidence**			
**Patient or population:** Policymakers and health system managers			
**Settings:**			
**Intervention:** evidence summaries based on systematic review			
**Comparison:** any comparison			
**Outcomes**	**Impact**	**No of Participants (studies)**	**Quality of the evidence (GRADE)**
**Use of systematic review evidence in decision‐making**	Little to no difference in effect on evidence‐informed decision‐making when compared to access to a knowledge broker or online registry of research.(Dobbins et al., 2009) Little to no difference in effect on self‐reported likelihood of using data‐driven versus story‐driven policy briefs (with state‐level or local‐level data).(Brownson et al., 2011)	399 (2)	⊕⊕⊕⊖**moderate** ^1^
**Understanding, knowledge and/or beliefs**	One study found little to no effect on understanding of information when provided in different Summary of Findings table formats (Vandvik et al., 2012)while the other found that those provided with a new version of the summary of findings table had consistently higher proportions of correct answers assessing understanding of key findings provided in the table.(Carrasco‐Labra et al., 2016) Little to no effect in understanding of information for a graded entry format compared to an summary of findings table or systematic review alone.(Opiyo et al., 2013) Little to no effect on changing participants' beliefs about the strength of the evidence for those who already had beliefs but increased the number of participants who had beliefs about the strength of the evidence.(Beynon et al., 2012; Masset et al., 2013)	676 (4)	⊕⊕⊕⊖**moderate** ^1^
**Perceived credibility of the summaries**	Little to no difference in perceived credibility for different versions of the policy brief (data‐driven versus story‐driven, local versus state‐level data).(Brownson et al., 2011)	291 (1)	⊕⊕⊕⊖**low** ^1,2^
**Perceived usefulness and usability of systematic review summaries**	The graded entry format was rated higher than the systematic review alone and there was little to no difference between the ratings for the summary of findings table and the systematic review alone.(Opiyo et al., 2013) Different summary of findings table formats had little to no effect in one study (Vandvik et al., 2012), but a new summary of findings format was found to be more accessible than the standard summary of findings in another.(Carrasco‐Labra et al., 2016)	443 (3)	⊕⊕⊕⊝**moderate^1^**
**Perceived understandability of the summaries**	All formats of the policy brief were reported as easy to understand.(Brownson et al., 2011) Graded entry formats were easier to understand the summary of findings tables or systematic reviews alone.(Opiyo et al., 2013)	356 (2)	⊕⊕⊕⊝**moderate^1^**
**Perceived desirability of the summaries**	Alternate versions of the summary of findings were preferred.(Carrasco‐Labra et al., 2016; Vandvik et al., 2012)	378 (2)	⊕⊕⊕⊝**moderate^1^**
GRADE Working Group grades of evidence **High quality:** Further research is very unlikely to change our confidence in the estimate of effect. **Moderate quality:** Further research is likely to have an important impact on our confidence in the estimate of effect and may change the estimate. **Low quality:** Further research is very likely to have an important impact on our confidence in the estimate of effect and is likely to change the estimate. **Very low quality:** We are very uncertain about the estimate.			
1. Unclear ROB 2. Only 1 study			

### Synthesis of results

#### Evidence of effectiveness

We generated a Summary of Findings table for this review (Table 4). This is a narrative summary of all studies assessing a particular outcome domain, grouped across different policy brief formats. We did not conduct a meta‐analysis because the interventions and outcomes of the included studies were too different to combine.

##### Primary outcomes: Use of summaries in decision‐making

Two studies assessed self‐reported use of summaries in decision‐making. First, Dobbins et al. assessed the change in global evidence‐informed decision‐making (EIDM) which is defined as the extent to which research evidence was considered in a decision 18 months after the intervention. The authors found that the intervention had no significant effect on EIDM. The post‐intervention change for the group receiving targeted, tailored messages was ‐0.42 (95% CI: ‐1; 0.26) and ‐0.09 (95% CI: ‐0.78, 0.60). This study also reported on evidence‐based public health policies and programs as a measure of the actual number of strategies, policies, and interventions for health body weight promotion among children that were implemented by the health department. For this outcome, the group that received the targeted, tailored messages had a statistically significant increase in evidence‐based public health policies and programs (post‐intervention change 1.67, 95% CI: 0.37, 2.97) compared with no change for the group with access to a knowledge broker (‐0.09, 95% CI (‐0.78, 0.60) (Dobbins et al., 2009).

The study by Brownson et al. asked policymakers how likely they would be to use the evidence summary in decision‐making. On a 5‐point Likert scale, where 1 is strongly disagree and 5 is strongly agree, there was little to no difference based on the type of policy brief (data‐driven versus story driven) (range 3.3 to 3.4). However, there were differences in self‐reported likelihood of using the policy brief depending on type of policymaker. Staff members reported being most likely to use the story‐focused brief with state‐level data (mean rating of 3.4, 95% confidence interval (CI) 3.0 to 3.9) and least likely to use the data‐focused brief with state‐level data (2.5, 95% CI 2.0 to 3.0). Legislators reported being most likely to use the data‐focused brief with state level data (4.1, 95% CI 3.6 to 4.6) and least likely to use story‐focused brief with state‐level data (3.1, 95% CI 2.6 to 3.6) (Brownson et al., 2011).

##### Understanding, knowledge, and/or beliefs

Carrasco‐Labra et al. found that respondents receiving the new summary of findings format had a higher proportion of correct answers for almost all questions. These included the ability to interpret footnotes (risk difference (RD) 7%, p=0.18), ability to determine risk difference (RD 63%, p=<0.001), understanding of quality of evidence and treatment effect (RD=62%, p=<0.001), understanding of the quality of evidence (RD 7%, p=0.06), and ability to quantify risk (RD 6%, p=0.06) (Carrasco‐Labra et al., 2016). However, for one question, the ability to relate the number of participants and studies to outcomes, the group receiving the standard summary of findings scored slightly higher (RD ‐3%, p=1.0).

The Masset study examined changes in beliefs about the effectiveness of the intervention as well as the strength of the evidence included in the policy briefs. The authors found that the policy brief increased the number of participants who had an opinion about the strength of the evidence. For example, those who did not have an opinion at baseline formed an opinion based on the policy brief. The difference‐in‐difference coefficients indicate that the policy brief increased the percentage of participants with an opinion about the strength of the evidence by 20–25 decimal points (Masset et al. 2013). However, the intervention was less effective in changing participants' ratings of the strength of the evidence or the effectiveness of the intervention (Masset et al., 2013). The policy brief did not change opinions of those who had an opinion at baseline about the evidence and effectiveness.

The Opiyo study found little to no difference between interventions for the odds of correct responses to questions about the intervention. The adjusted odds ratio (OR) for the summary of findings table compared to the systematic review alone was 0.59(95% CI 0.32 to 1.07), and graded entry format compared to systematic review alone was 0.66 (95% CI 0.36 to 1.21). However, both of these indicated that the odds of correct responses were higher for the groups who received an evidence summary or summary of findings (Opiyo et al., 2013). When comparing groups of participants, both the summary of findings tables and the graded entry formats slightly improved understanding for policymakers. For the summary of findings table compared to systematic review alone, the adjusted OR was 1.5 (95% CI 0.15‐15.15) and the graded entry format compared to systematic review alone was 1.5 (0.64‐3.54) (Opiyo et al., 2013).

Finally, Vandvik et al. reported that there was little to no difference in participants' understanding of information in the different table formats for most items (range 80% to 97% for table A compared to 69% to 92% for table B, p‐values from 0.26 to 0.86) However, more participants with table A had correct answers for two items: time period for risk estimates (58% correct compared to 11% correct, p<0.0001) and the range in which the effect may lie (95% correct versus 54% correct, p<0.0001) (Vandvik et al., 2012).

##### Secondary outcomes: Credibility of the summaries

Brownson et al. reported little to no differences in credibility for the different intervention formats (low certainty of evidence). Mean scores for perceived credibility ranged from 4.4 to 4.5 on a 5‐point Likert scale in which 5 indicated “strongly agree” (Brownson et al., 2011). For different policymaker groups there were also little to no differences with mean scores ranging from 4.2 to 4.5 for staff members, 4.3 to 4.7 for legislators, and 4.3 to 4.6 for executives (Brownson et al., 2011).

##### Perceived usefulness and usability of the summaries

The Carrasco‐Labra study reported that the new summary of findings format was more accessible than the standard format (Carrasco‐Labra et al., 2016). This was assessed using a 7‐point scale for which 1 indicates “strongly disagree” and 7 indicates “strongly agree”. Participants who received the new SOF format reported that it was easy to find the information about the effects more often (adjusted mean difference (MD) 0.4, SE 0.19, p=0.04, representing a 5.7% difference) and easy to understand the information more often (MD 0.5, SE 0.2, p=0.011, representing a 7.1% difference). The respondents also reported that the new format displayed results in a way that was more helpful for decision‐making (MD 0.5, SE 0.18, p=0.011, 7.1% difference).

Opiyo et al. measured this outcome by assessing the ‘value and accessibility’ of each intervention. The graded entry format received a higher mean score than the systematic review alone (MD 0.52 (95% CI 0.06 to 0.99). The odds of a one‐point increase for the graded entry format compared to the systematic review alone were 1.52 (95% CI: 1.06‐2.20). There was little to no difference in effect when comparing the summary of findings table and the systematic review alone (MD ‐0.11, 95% CI ‐0.71 to 0.48). The odds of a one‐point increase were 0.91 (95% CI: 0.57‐1.46) (Opiyo et al., 2013).

Vandvik et al. reported that accessibility of information for quality of evidence as well as absolute and relative effects was rated similarly with no significant differences between groups (Vandvik et al., 2012). Only pooled results were presented.

##### Perceived understandability of the summaries

All the groups in the Brownson et al. study reported that the summaries were easy to understand (Brownson et al., 2011). Mean ratings ranged from 4.3 to 4.4 on a 5‐point Likert scale. For the different policymaker groups, there was little to no difference with mean scores ranging from 4.3 to 4.5 for staff members and legislators and 4.1 to 4.4 for executives (Brownson et al., 2011).

The study by Opiyo et al. reported that 60% (95% CI: 48% to 73%) of the participants found systematic reviews to be more difficult to read than the narrative reports included in the graded entry formats. Fifty‐one percent of participants (95% CI: 38% to 63%) found systematic reviews to be easier to read compared to 26% (95% CI: 15% to 37%) who found summary of findings tables easier. 53% (95% CI: 41% to 65%) of participants preferred the narrative report format (graded entry) compared to 25% (95% CI: 14% to 36%) who preferred the full systematic review (Opiyo et al., 2013). The majority of participants interviewed reported that narrative formats were clearer, easy to read, and easy to understand and some participants reported that the Summary of Findings tables were difficult to understand as a stand‐alone product.

##### Perceived desirability of the summaries

Two studies of different summary of findings formats assessed this outcome. One study found that participants preferred the summary of findings table which presented study event rates versus the one without them (median 1, interquartile range (IQR) 1, on 1‐7 scale in which 1 indicates ‘strong preference for’ and 7 indicates ‘strong preference against’). Participants preferred the table with the absolute risk differences versus presentation of absolute risks only (median 2, IQR 3). Additional information embedded in table was preferred versus having it as footnotes (median 1, IQR 2). No significant differences were found for the placement of the column for ‘overall quality of evidence’ either as the final column or before the effect size or the overall table format (Vandvik et al., 2012).

The other study found that overall, respondents preferred the new summary of findings format (MD 2.8, SD 1.6) compared to the standard format (Carrasco‐Labra et al., 2016).

None of the included studies reported on policymakers' perceived relevance of the summaries.

##### Effect modifiers

The organizational research culture was found to influence the effect of the intervention on evidence‐based public health policies and programs in one study which found that tailored, targeted messages were more effective than access to a database alone (healthevidence.org) or access to a knowledge broker when the organization valued research evidence in decision‐making (Dobbins et al., 2009). In this study, organizational culture referred to characteristics such as the value placed on research in decision‐making, the expectation of the use of research in decision‐making, and staff training in research methods and critical appraisal and was assessed on a seven point scale.

The Carrasco‐Labra study conducted regression analyses to assess potential effect modifiers, such as the number of years of experience in guideline development, as a researcher or as a health care provider. They found that the number of years of experience modified the effect on understanding by more than 10% (adjusted OR 1.83; 95% CI 0.91 to 3.67) for the questions about the ability to determine a risk difference. For the question assessing whether respondents understand the quality of evidence and treatment effect combined, the authors found that years of experience, familiarity with GRADE, and level of training modified the effect by more than 10% (adjusted OR 0.72; 95% CI 0.20 to 2.56) (Carrasco‐Labra et al., 2016).

## Discussion

### Summary of main results

This review has summarized the evidence on the use of systematic review summaries in policy‐making, policy‐makers' understanding of systematic review evidence, and different components and design features. Overall, the results suggest that evidence summaries may be easier to understand than complete systematic reviews. However, their ability to increase the use of systematic review evidence in policymaking is unclear because not enough evidence is available.

Six studies were included in this review. For our primary outcome, use of systematic review evidence in decision‐making, one study found that targeted, tailored messages increased the number of evidence‐based public health policies and programs. However, for the two studies that assessed effect on decision‐making or likelihood of using the summary in decision‐making there was little to no difference between intervention groups (Brownson et al., 2011; Dobbins et al., 2009). We assessed these results as having moderate certainty using GRADE.

For the secondary outcome of understanding, knowledge, and beliefs, there was little to no difference in effect and moderate certainty evidence in three of the four studies assessing this outcome (Masset et al., 2013; Opiyo et al., 2013; Vandvik et al., 2012). There was a slight increase in understanding for summary of findings tables and graded entry formats compared to systematic reviews alone. The fourth study found that participants provided with an alternate version of the summary of findings table had greater understanding (Carrasco‐Labra et al., 2016).

For perceived desirability of summaries we found moderate certainty of evidence. One study found that the alternate version of the summary of findings table was preferred, (Carrasco‐Labra et al., 2016) and the other study found that certain formatting elements such as study event rates, and absolute risk differences were preferred as well as additional information provided in the table and not in footnotes (Vandvik et al., 2012). One study found the alternate format to be more accessible than the standard format, (Carrasco‐Labra et al., 2016) however, the other study assessing formatting changes found little to no difference in effect for perceived usefulness (Vandvik et al., 2012).

For perceived usefulness and usability of the summaries, we found moderate certainty evidence. The graded entry summary was rated higher than a systematic review alone for usability (Opiyo et al., 2013). Summaries were perceived as easier to understand than systematic reviews (moderate certainty evidence) (Brownson et al., 2011; Opiyo et al., 2013). There was little to no difference in effect for different versions of the policy brief (data‐driven versus story‐driven, local versus state‐level data; moderate certainty evidence) for perceived credibility of the summaries (Brownson et al., 2011).

### Overall completeness and applicability of evidence

We identified two protocols for ongoing studies which is promising as the results of these studies will enhance the available evidence about the effectiveness of evidence summaries (Wilson et al., 2011; Wilson et al., 2015). We also identified other relevant studies assessing the effectiveness of systematic review derivatives that did not use an eligible study design (e.g. used interviews or other methods without a control group) (Rosenbaum et al., 2011; Wilson et al., 2015). One of these studies was intended to be a RCT and process evaluation but was not eligible for our review because poor recruitment (only 15% of the planned sample) resulted in the termination of the trial (Wilson et al., 2015). This demonstrates the difficulty with recruiting these types of participants. Recruitment for the process evaluation remained low and the authors noted that those included are likely those already more interested in using systematic review derivatives (Wilson et al., 2015). The authors noted that, for future RCTs, recruitment may be more successfully achieved from randomizing divisions versus individuals because the nature of policymaking is quite complex and often not completed at the individual level. Additionally, we identified other studies that were not focused on policymakers but rather clinicians (Noor, 2009; Laure Perrier et al., 2015) or the public (Oermann et al., 2009). These studies demonstrated that evidence summaries can improve understanding of research evidence within these populations however use of evidence in decision‐making was not assessed.

### Quality of the evidence

We used the GRADE approach to assess the overall quality or certainty of the evidence included in this review. We assessed most of the evidence to be ‘moderate’ certainty. For perceived desirability of the summaries, we assess the evidence as ‘high’ certainty.

### Limitations and potential biases in the review process

Our primary outcome, policymakers' use of systematic review evidence in decision‐making is challenging to measure. Other studies have noted the inherent challenges in measuring this outcome because many factors contribute to decision‐making and it is often difficult for an individual to identify which factors had a role in their final decision (Dobbins et al., 2009; Dobbins et al., 2007). Instead of determining the actual use of research in decision‐making, studies assessed self‐reported use of research or other outcomes, such as perceived credibility or relevance since these may affect the likelihood of research use in decision‐making.

Our primary outcome was policymakers' use of evidence and therefore we restricted to studies that would provide a quantitative measure. We therefore limited inclusion to RCTs, NRCTs, CBA studies, and ITS studies but planned to extract qualitative data for these outcomes, when provided. Only one study presented qualitative data and and five studies were excluded because they were qualitative studies. These studies may provide additional data regarding the credibility, desirability, relevance, understandability, and usefulness of evidence summaries which would be helpful for understanding how and why evidence summaries could be useful in policymaking.

We did not assess the use of health behaviour theory. This may be an important concept for understanding the effectiveness of evidence summaries and therefore we plan to include it in future updates of this review.

Our review is limited by the indexing of studies in this area. To address this issue, we conducted a broad search using search strategies adapted from similar systematic review. Our search identified over 10,000 references but we had a low yield of included studies. The methods used in the included studies were poorly reported. For example, only two studies adequately reported on random sequence generation or allocation concealment, which means that most studies have unclear risk of bias.

### Agreements and disagreements with other studies or reviews

Previous systematic reviews have assessed the effectiveness of interventions for improving the use of systematic reviews in decision‐making by health system managers, policymakers, and clinicians (Murthy et al., 2012); interventions encouraging the use of systematic reviews by health policymakers and managers (Perrier et al., 2011); and facilitators to the uptake of systematic review evidence (Wallace et al., 2012).

Murthy et al. assessed the effectiveness of interventions for improving the use of systematic reviews in decision‐making by health system managers, policymakers, and clinicians (Murthy et al., 2012). Similar to our findings, they found that evidence presented as a single, clear message may improve evidence‐based practice (Murthy et al., 2012). The review by Perrier et al. found little evidence on interventions that encourage the use of systematic reviews by policymakers and managers (Perrier et al., 2011).

The review by Wallace et al. assessed potential facilitators to the use of systematic review evidence by policy‐makers and identified five potential factors: the perception that systematic reviews may be used for improving knowledge, research, and evidence‐based medicine skills, content that assesses benefits, harms and costs and is perceived as current,

transparent and timely; a ‘graded entry’ format; training in use of systematic reviews and peer‐group support (Wallace et al., 2012). We did not assess facilitators to the use of systematic review evidence in our review; however, we did find that the ‘graded entry’ format may be easier to understand than complete systematic reviews.

## Authors' conclusions

The interventions assessed in the studies included in our review are quite diverse with a variety of outcome measures. We included a broad range of interventions to provide an overview of the evidence on systematic review derivative products. These products have important differences in design and source material. For example, a policy brief includes evidence from one or more systematic reviews and includes information from additional sources (Adam et al., 2014; Moat et al., 2013) whereas a summary of findings table reports results for a single systematic review. We chose to include all systematic review derivative products as there are limited studies on a single product type. We recognize that this creates a challenge for interpreting the results because the interventions were quite different. Therefore, we have provided a narrative summary of each study and presented an overview of the available evidence.

### Implications for practice and policy

Overall, the studies included in our review suggest that evidence summaries may be easier to understand than complete systematic reviews. To facilitate the use of systematic review evidence by policymakers, reviews need to be perceived to be of high quality and assess a relevant research question (Oliver et al., 2014). Systematic reviews and their derivative products should be developed with collaboration from their intended users.

### Implications for research

Future studies should include an assessment of delivery strategies because the effectiveness of the systematic review derivative product in practice will be affected by policymakers' knowledge of and access to the summaries themselves. Our included studies suggest that evidence summaries have a small effect on improving knowledge and understanding and may be easier for policymakers to understand. However, we have very little evidence to inform the design of evidence summaries because we only found a handful of different formats (none the same), and there was little to no difference between formats when compared directly.

It is important to note that only two of the included studies compared the evidence summary to a full systematic review or access to a database of systematic reviews. The others compared different versions of evidence summaries and, in general, found little to no differences in the effects. Had these studies included systematic reviews as a control group the results may be different.

Future studies should ensure that derivative products are compared to complete systematic reviews. Qualitative methods may be appropriate for future evaluations to assess the effectiveness, credibility, desirability, understandability, usefulness, and relevance of evidence summaries. Similarly, future reviews may consider qualitative synthesis which may add a deeper level of understanding to our findings.

Additional research on the use of evidence summaries derived from systematic reviews is needed. Researchers should consider the primary goal of these derivative products, which is to increase the uptake of systematic review evidence, and aim to assess this in future studies. The production of evidence summaries and other systematic review derivative products is increasing and therefore, more evaluation of their effectiveness on increasing the use of systematic reviews by policymakers and health system managers is needed to ensure they have the desired effect of increasing the use of systematic review evidence in decision‐making.

## Information about this review

### Review authors

#### Lead review author

The lead author is the person who develops and co‐ordinates the review team, discusses and assigns roles for individual members of the review team, liaises with the editorial base and takes responsibility for the on‐going updates of the review.

**Table:** 

**Name:** Jennifer Petkovic
Affiliation: University of Ottawa
Address: 85 Primrose Ave
City, State, Province or County: Ottawa, Ontario
Post code: K1N 6M1
Country: Canada
Phone: 613‐562‐6262 ext. 2905
Email: jennifer.petkovic@uottawa.ca
**Co‐authors**
**Name:** Vivian Welch
Affiliation: Bruyère Research Institute
Address:85 Primrose Ave
City, State, Province or County: Ottawa, Ontario
Post code: K1N6M1
Country: Canada
Email: Vivian.welch@uottawa.ca
**Name:** Maria Helena Jacob
Affiliation: Bruyere Research Institute
Address: 85 Primrose Ave.
City, State, Province or County: Ottawa, Ontario
Post code: K1N6M1
Country: Canada
Email: mhvjacob@hotmail.com
**Name:** Manosila Yoganathan
Affiliation: Bruyère Research Institute
Address: 85 Primrose Ave.
City, State, Province or County: Ottawa, Ontario
Post code: K1N6M1
Country: Canada
Email: y.manosilah@gmail.com
**Name:** Ana Patricia Ayala
Affiliation: Gerstein Science Information Centre, University of Toronto
City, State, Province or County: Toronto, Ontario
Country: Canada
Email: anap.ayala@utoronto.ca
**Name:** Heather Cunningham
Affiliation: Gerstein Science Information Centre, University of Toronto
City, State, Province or County: Toronto, Ontario
Country: Canada
Email: h.cunningham@utoronto.ca
**Name:** Peter Tugwell
Affiliation: University of Ottawa
City, State, Province or County: Ottawa, Ontario
Country: Canada
Email: tugwell.bb@uottawa.ca

### Roles and responsibilities

- Content: Jennifer Petkovic, Peter Tugwell, Vivian Welch
- Systematic review methods: Jennifer Petkovic, Peter Tugwell, Vivian Welch
- Statistical analysis: Jennifer Petkovic, Vivian Welch
- Information retrieval: Ana Patricia Ayala, Heather Cunningham, Manosila Yoganathan

### Acknowledgements

The authors thank Christian Charbonneau who assisted with the screening and data extraction.

### Sources of support

This work was supported by JPs CIHR Doctoral Research Award and Campbell Systematic Review Award.

### Declarations of interest

None to declare.

### Plans for updating the review

Jennifer Petkovic will be responsible for ensuring that this review is kept up‐to‐date.

## Supporting information

Supplementary material
