# Supplementary material for: Do evidence summaries increase health policy‐makers' use of evidence from systematic reviews? A systematic review
Source: Campbell Syst Rev. 2018 Sep 10;14(1):1–52. doi: 10.4073/csr.2018.8 (PMC8428003; doi:10.4073/csr.2018.8)
Supplement: Supplementary file 1 — Supplementary material [file CL2-14--s001.docx]

# Online supplements

Do evidence summaries increase health policy-makers’ use of evidence from systematic reviews? A systematic review

List of online supplements

1. Online supplement 1: Bibliographic databases search strategies

2. Online supplement 2: Grey literature sources

3. Online supplement 3: Excluded studies

4. Online supplement 4: Risk of bias assessments

**Online Supplement 1: Bibliographic databases search strategies**

**Database: Ovid MEDLINE(R) In-Process & Other Non-Indexed Citations and Ovid MEDLINE(R) <1946 to Present>**

Search Strategy:

--------------------------------------------------------------------------------

1 ((systematic review$ or methodolog$ review$ or quantitativ$ review$ or qualitativ$ review$ or overview$ or synthes$ or metasynthes$ or megasynthes$) adj5 (decisionmak$ or decision-mak$ or policy-mak$ or policymak$ or policy decision$ or health$ polic$ or health$ manag$ or action$ or commission* or purchas* or procur* or budget hold* or budgethold* or service provi*or practice or application or implement$ or utili?ation or utili?ing or utili$ or disseminat$ or summar$ or hospital* decision* or treatment plan* or patient care or patientcare or healthcare or health care or clinical decision* or pathway* or algorithm*)).ti,ab. (30531)

2 (((systematic adj2 (review* or overview* or synthesis or literature review* or evidence review*)) or methodolog* review* or quantitativ* review* or qualitative review* or overview or synthes* or metasynthes* or megasynthes*) adj5 (policy or policies or decision*)).ti. (400)

3 ((gap or gaps) adj7 ((knowledge or research or evidence or trial or result) adj2 practice)).ti,ab. (972)

4 1 or 2 or 3 (31697)

5 randomized controlled trial.pt. (409861)

6 controlled clinical trial.pt. (90286)

7 randomized.ab. (339312)

8 placebo.ab. (167495)

9 clinical trials as topic/ (175364)

10 randomly.ab. (244542)

11 trial.ti. (147363)

12 intervention*.ti. (96258)

13 or/5-12 (1074398)

14 State Medicine/ (52025)

15 exp Purchasing, Hospital/ (5754)

16 Contracts/ (2870)

17 exp Contract Services/ (12176)

18 exp Organizational Innovation/ (23682)

19 Insurance, Health/ or exp Managed Care Programs/ or Medicare/ (96098)

20 (commissioning or commissioner$).ti,ab. (4202)

21 (purchasing or purchaser$).ti,ab. (8743)

22 (procurement or procurer$).ti,ab. (6959)

23 (budget-holder$ or budgetholder$).ti,ab. (48)

24 (service adj2 (development or developer$ or provision or provider$)).ti,ab. (12067)

25 ((investment or budget or purchas$ or service) adj3 priorit$).ti,ab. (554)

26 priorit$ setting.ti,ab. (1563)

27 decision-maker$.ti,ab. (9028)

28 (contract$ adj3 (management or services or tender$)).ti,ab. (1293)

29 Decision Making, Organizational/ (10711)

30 exp Policy Making/ (20784)

31 exp Health Planning/ (297761)

32 or/14-31 (454534)

33 exp Evidence-Based Practice/ (68838)

34 Translational Research/ (6166)

35 exp "Diffusion of Innovation"/ (16708)

36 ((research or knowledge or innovation$ or evidence) adj5 (diffus$ or disseminat$ or implement$ or adoption or exchang$ or application or mobilis$ or mobiliz$ or synthes$ or transfer$ or translat$ or incorporat$ or uptak$ or utilis$ or utiliz$ or transmission or integrat$ or democratis$ or democratiz$ or shar$ or broke$)).ti,ab. (105758)

37 ('research into practice' or 'knowledge into practice' or 'knowledge into action' or 'research into action' or 'research findings into action' or 'evidence into action' or 'evidence into practice').ti,ab. (1564)

38 (KT adj5 (diffus$ or disseminat$ or implement$ or adoption or exchang$ or application or mobilis$ or mobiliz$ or synthes$ or transfer$ or translat$ or incorporat$ or uptak$ or utilis$ or utiliz$ or transmission or integrat$ or democratis$ or democratiz$ or shar$ or broke$)).ti,ab. (594)

39 ((evidence base$ or evidence inform$) adj5 (decision$ or plan$ or policy or policies or practice or action$)).ti,ab. (15612)

40 ((research or knowledge or innovation$ or evidence) adj5 (change$ or changing or improv$ or promot$ or influenc$ or impact$ or disinvest$ or discontinu$ or reject$ or abandon$ or ceas$ or restrict$ or disincentiv$ or stop$)).ti,ab. (116242)

41 ((research utiliz$ or research utilis$ or evidence or knowledge or innovation$) adj5 (decision-mak$ or decisionmak$ or policy-mak$ or policymak$ or health$ manag$ or health$ polic$ or action$ or practice or policy decision$)).ti,ab. (39062)

42 (('use' or using or usage or useful or utiliz$ or utilis$) adj5 (evidence or research)).ti,ab. (83100)

43 Information Dissemination/ (11958)

44 (disseminat$ adj5 (findings or results)).ti,ab. (3346)

45 'Health Knowledge, Attitudes, Practice'/ (0)

46 Attitude of Health Personnel/ (98662)

47 Clinical Competence/ (71888)

48 or/34-47 (482218)

49 ((research or knowledge or innovation$ or evidence or information or policy) adj5 (brief$ or summar$ or synops$ or overview$ or bulletin$ or synthes$ or map or mapping or maps or framing$ or product$ or package$ or alert$ or commentar$ or strateg$ or algorithm$)).ti,ab. (116091)

50 (push activit* or pull activit*).ti,ab. (4)

51 (collaborat$ or 'cross-profession$' or intraprofession$ or intra-profession$ or interprofession$ or inter-profession$ or inter-disciplin$ or multi-disciplin$ or multi disciplin$ or multiprofession$ or outsourc$ or subcontract$).ti,ab. (106128)

52 'linkage.mp. and exchange'.ti,ab. [mp=title, abstract, original title, name of substance word, subject heading word, keyword heading word, protocol supplementary concept word, rare disease supplementary concept word, unique identifier] (1725)

53 or/48-51 (655194)

54 4 and 13 and (32 or 48 or 53) (867)

55 intervention?.ti. or (intervention? adj6 (clinician? or collaborat$ or community or complex or DESIGN$ or doctor? or educational or family doctor? or family physician? or family practitioner? or financial or GP or general practice? or hospital? or impact? or improv$ or individuali?e? or individuali?ing or interdisciplin$ or multicomponent or multi-component or multidisciplin$ or multi-disciplin$ or multifacet$ or multi-facet$ or multimodal$ or multi-modal$ or personali?e? or personali?ing or pharmacies or pharmacist? or pharmacy or physician? or practitioner? or prescrib$ or prescription? or primary care or professional$ or provider? or regulatory or regulatory or tailor$ or target$ or team$ or usual care)).ab. (195610)

56 (pre-intervention? or preintervention? or "pre intervention?" or post-intervention? or postintervention? or "post intervention?").ti,ab. (13214)

57 demonstration project?.ti,ab. (2113)

58 (pre-post or "pre test$" or pretest$ or posttest$ or "post test$" or (pre adj5 post)).ti,ab. (78828)

59 (pre-workshop or post-workshop or (before adj3 workshop) or (after adj3 workshop)).ti,ab. (738)

60 trial.ti. or ((study adj3 aim?) or "our study").ab. (766742)

61 (before adj10 (after or during)).ti,ab. (393848)

62 ("quasi-experiment$" or quasiexperiment$ or "quasi random$" or quasirandom$ or "quasi control$" or quasicontrol$ or ((quasi$ or experimental) adj3 (method$ or study or trial or design$))).ti,ab,hw. (115533)

63 ("time series" adj2 interrupt$).ti,ab,hw. (1414)

64 (time points adj3 (over or multiple or three or four or five or six or seven or eight or nine or ten or eleven or twelve or month$ or hour? or day? or "more than")).ab. (11325)

65 pilot.ti. (47215)

66 Pilot projects/ (91909)

67 (clinical trial or controlled clinical trial or multicenter study).pt. (661665)

68 (multicentre or multicenter or multi-centre or multi-center).ti. (34249)

69 random$.ti,ab. or controlled.ti. (863821)

70 (control adj3 (area or cohort? or compare? or condition or design or group? or intervention? or participant? or study)).ab. not (controlled clinical trial or randomized controlled trial).pt. (470356)

71 evaluation studies as topic/ or prospective studies/ or retrospective studies/ (1072256)

72 (utili?ation or programme or programmes).ti. (60938)

73 (during adj5 period).ti,ab. (328074)

74 ((strategy or strategies) adj2 (improv$ or education$)).ti,ab. (22557)

75 "comment on".cm. or review.pt. or (review not "peer review$").ti. or randomized controlled trial.pt. (3239935)

76 (rat or rats or cow or cows or chicken? or horse or horses or mice or mouse or bovine or animal?).ti. (1429818)

77 exp animals/ not humans.sh. (4203554)

78 (or/55-74) not (or/75-77) (2709421)

79 4 and 78 and (32 or 48 or 53) (615)

**2. Web of Science (3671)**

| Set | Results |  |
| --- | --- | --- |
| # 42 | [3,639](http://apps.webofknowledge.com.proxy.bib.uottawa.ca/summary.do?product=WOS&doc=1&qid=51&SID=3FHSDaBySDxR4AEIsk3&search_mode=CombineSearches&update_back2search_link_param=yes) | #41 AND #40  *Indexes=SCI-EXPANDED, SSCI, A&HCI, CPCI-S, CPCI-SSH, ESCI Timespan=All years* |
| # 41 | [13,170](http://apps.webofknowledge.com.proxy.bib.uottawa.ca/summary.do?product=WOS&doc=1&qid=50&SID=3FHSDaBySDxR4AEIsk3&search_mode=AdvancedSearch&update_back2search_link_param=yes) | #4 AND (#39 OR #34 OR #20)  *Indexes=SCI-EXPANDED, SSCI, A&HCI, CPCI-S, CPCI-SSH, ESCI Timespan=All years* |
| # 40 | [2,735,585](http://apps.webofknowledge.com.proxy.bib.uottawa.ca/summary.do?product=WOS&doc=1&qid=41&SID=3FHSDaBySDxR4AEIsk3&search_mode=GeneralSearch&update_back2search_link_param=yes) | TOPIC: ((randomized or randomised or controlled trial* or Random A llocation or Double-Blind or Single-Blind or Clinical Trial* or comparative stud* or intervention stud* or control group* or placebo* or evaluation stud* or placebo*))  *Indexes=SCI-EXPANDED, SSCI, A&HCI, CPCI-S, CPCI-SSH, ESCI Timespan=All years* |
| # 39 | [520,471](http://apps.webofknowledge.com.proxy.bib.uottawa.ca/summary.do?product=WOS&doc=1&qid=40&SID=3FHSDaBySDxR4AEIsk3&search_mode=CombineSearches&update_back2search_link_param=yes) | #38 OR #37 OR #36 OR #35  *Indexes=SCI-EXPANDED, SSCI, A&HCI, CPCI-S, CPCI-SSH, ESCI Timespan=All years* |
| # 38 | [15](http://apps.webofknowledge.com.proxy.bib.uottawa.ca/summary.do?product=WOS&doc=1&qid=39&SID=3FHSDaBySDxR4AEIsk3&search_mode=GeneralSearch&update_back2search_link_param=yes) | TOPIC: ((“linkage and exchange”))  *Indexes=SCI-EXPANDED, SSCI, A&HCI, CPCI-S, CPCI-SSH, ESCI Timespan=All years* |
| # 37 | [237,062](http://apps.webofknowledge.com.proxy.bib.uottawa.ca/summary.do?product=WOS&doc=1&qid=38&SID=3FHSDaBySDxR4AEIsk3&search_mode=GeneralSearch&update_back2search_link_param=yes) | TOPIC: (((collaborat* or “cross-profession*” or intraprofession* or “intra-profession*” or interprofession* or “inter-profession*” or “inter-disciplin*” or “multi-disciplin*” or “multi discipline*” or multiprofession* or outsource* or subcontract*)))  *Indexes=SCI-EXPANDED, SSCI, A&HCI, CPCI-S, CPCI-SSH, ESCI Timespan=All years* |
| # 36 | [16](http://apps.webofknowledge.com.proxy.bib.uottawa.ca/summary.do?product=WOS&doc=1&qid=37&SID=3FHSDaBySDxR4AEIsk3&search_mode=GeneralSearch&update_back2search_link_param=yes) | TOPIC: ((("push activit*" or "pull activit*")))  *Indexes=SCI-EXPANDED, SSCI, A&HCI, CPCI-S, CPCI-SSH, ESCI Timespan=All years* |
| # 35 | [294,046](http://apps.webofknowledge.com.proxy.bib.uottawa.ca/summary.do?product=WOS&doc=1&qid=36&SID=3FHSDaBySDxR4AEIsk3&search_mode=GeneralSearch&update_back2search_link_param=yes) | TOPIC: ((((research or knowledge or innovation* or evidence or information or policy) near/5 (brief$ or summar* or synops* or overview* or bulletin* or synthes* or map or mapping or maps or framing* or product* or package* or alert* or commentar* or strategy* or algorithm*))))  *Indexes=SCI-EXPANDED, SSCI, A&HCI, CPCI-S, CPCI-SSH, ESCI Timespan=All years* |
| # 34 | [763,235](http://apps.webofknowledge.com.proxy.bib.uottawa.ca/summary.do?product=WOS&doc=1&qid=35&SID=3FHSDaBySDxR4AEIsk3&search_mode=CombineSearches&update_back2search_link_param=yes) | #33 OR #32 OR #31 OR #30 OR #29 OR #28 OR #27 OR #26 OR #25 OR #24 OR #23 OR #22 OR #21  *Indexes=SCI-EXPANDED, SSCI, A&HCI, CPCI-S, CPCI-SSH, ESCI Timespan=All years* |
| # 33 | [3,402](http://apps.webofknowledge.com.proxy.bib.uottawa.ca/summary.do?product=WOS&doc=1&qid=34&SID=3FHSDaBySDxR4AEIsk3&search_mode=GeneralSearch&update_back2search_link_param=yes) | TOPIC: ((“clinical competenc*” or “clinical skill$”))  *Indexes=SCI-EXPANDED, SSCI, A&HCI, CPCI-S, CPCI-SSH, ESCI Timespan=All years* |
| # 32 | [30,591](http://apps.webofknowledge.com.proxy.bib.uottawa.ca/summary.do?product=WOS&doc=1&qid=33&SID=3FHSDaBySDxR4AEIsk3&search_mode=GeneralSearch&update_back2search_link_param=yes) | TOPIC: ((health near/2 (knowledge or attitudes or practice)))  *Indexes=SCI-EXPANDED, SSCI, A&HCI, CPCI-S, CPCI-SSH, ESCI Timespan=All years* |
| # 31 | [14,819](http://apps.webofknowledge.com.proxy.bib.uottawa.ca/summary.do?product=WOS&doc=1&qid=32&SID=3FHSDaBySDxR4AEIsk3&search_mode=GeneralSearch&update_back2search_link_param=yes) | TOPIC: (((disseminat* near/5 (findings or results or information))))  *Indexes=SCI-EXPANDED, SSCI, A&HCI, CPCI-S, CPCI-SSH, ESCI Timespan=All years* |
| # 30 | [219,358](http://apps.webofknowledge.com.proxy.bib.uottawa.ca/summary.do?product=WOS&doc=1&qid=31&SID=3FHSDaBySDxR4AEIsk3&search_mode=GeneralSearch&update_back2search_link_param=yes) | TOPIC: (((('”use”' or using or usage or useful or utilize* or utilis*) near/5 (evidence or research))))  *Indexes=SCI-EXPANDED, SSCI, A&HCI, CPCI-S, CPCI-SSH, ESCI Timespan=All years* |
| # 29 | [71,966](http://apps.webofknowledge.com.proxy.bib.uottawa.ca/summary.do?product=WOS&doc=1&qid=30&SID=3FHSDaBySDxR4AEIsk3&search_mode=GeneralSearch&update_back2search_link_param=yes) | TOPIC: ((((“research utilize*” or “research utilis*” or evidence or knowledge or innovation$) near/5 (“decision-mak*” or decisionmak* or “policy-mak*” or policymak* or “health* manag*” or “health* polic*” or action* or practice or “policy decision*”))))  *Indexes=SCI-EXPANDED, SSCI, A&HCI, CPCI-S, CPCI-SSH, ESCI Timespan=All years* |
| # 28 | [241,292](http://apps.webofknowledge.com.proxy.bib.uottawa.ca/summary.do?product=WOS&doc=1&qid=29&SID=3FHSDaBySDxR4AEIsk3&search_mode=GeneralSearch&update_back2search_link_param=yes) | TOPIC: ((((research or knowledge or innovation$ or evidence) near/5 (change$ or changing or improve* or promot* or influenc* or impact* or disinvest* or discontinue* or reject* or abandon* or ceas* or restrict* or disincentive* or stop*))))  *Indexes=SCI-EXPANDED, SSCI, A&HCI, CPCI-S, CPCI-SSH, ESCI Timespan=All years* |
| # 27 | [19,947](http://apps.webofknowledge.com.proxy.bib.uottawa.ca/summary.do?product=WOS&doc=1&qid=28&SID=3FHSDaBySDxR4AEIsk3&search_mode=GeneralSearch&update_back2search_link_param=yes) | TOPIC: ((((“evidence base*” or “evidence inform*”) near/5 (decision* or plan* or policy or policies or practice or action$))))  *Indexes=SCI-EXPANDED, SSCI, A&HCI, CPCI-S, CPCI-SSH, ESCI Timespan=All years* |
| # 26 | [698](http://apps.webofknowledge.com.proxy.bib.uottawa.ca/summary.do?product=WOS&doc=1&qid=27&SID=3FHSDaBySDxR4AEIsk3&search_mode=GeneralSearch&update_back2search_link_param=yes) | TOPIC: (((KT near/5 (diffuse* or disseminat* or implement* or adoption or exchang* or application or mobilis* or mobiliz* or synthes* or transfer* or translat* or incorporate* or uptake* or utilis* or utilize* or transmission or integrat* or democratis* or democratiz* or shar* or broke*))))  *Indexes=SCI-EXPANDED, SSCI, A&HCI, CPCI-S, CPCI-SSH, ESCI Timespan=All years* |
| # 25 | [1,629](http://apps.webofknowledge.com.proxy.bib.uottawa.ca/summary.do?product=WOS&doc=1&qid=26&SID=3FHSDaBySDxR4AEIsk3&search_mode=GeneralSearch&update_back2search_link_param=yes) | TOPIC: (((“research into practice” or “knowledge into practice” or “knowledge into action” or “research into action” or “research findings into action” or “evidence into action” or “evidence into practice”)))  *Indexes=SCI-EXPANDED, SSCI, A&HCI, CPCI-S, CPCI-SSH, ESCI Timespan=All years* |
| # 24 | [262,137](http://apps.webofknowledge.com.proxy.bib.uottawa.ca/summary.do?product=WOS&doc=1&qid=25&SID=3FHSDaBySDxR4AEIsk3&search_mode=GeneralSearch&update_back2search_link_param=yes) | TOPIC: ((((research or knowledge or innovation$ or evidence) near/5 (diffuse* or disseminat* or implement* or adoption or exchang* or application or mobilis* or mobiliz* or synthes* or transfer* or translat* or incorporate* or uptake* or utilis* or utilize* or transmission or integrat* or democratis* or democratiz* or shar$ or broke*))))  *Indexes=SCI-EXPANDED, SSCI, A&HCI, CPCI-S, CPCI-SSH, ESCI Timespan=All years* |
| # 23 | [13,013](http://apps.webofknowledge.com.proxy.bib.uottawa.ca/summary.do?product=WOS&doc=1&qid=24&SID=3FHSDaBySDxR4AEIsk3&search_mode=GeneralSearch&update_back2search_link_param=yes) | TOPIC: (((diffusion near/2 innovation) or “technology transfer”))  *Indexes=SCI-EXPANDED, SSCI, A&HCI, CPCI-S, CPCI-SSH, ESCI Timespan=All years* |
| # 22 | [8,717](http://apps.webofknowledge.com.proxy.bib.uottawa.ca/summary.do?product=WOS&doc=1&qid=23&SID=3FHSDaBySDxR4AEIsk3&search_mode=GeneralSearch&update_back2search_link_param=yes) | TOPIC: ((translational near/1 (research or medicine or medical or knowledge)))  *Indexes=SCI-EXPANDED, SSCI, A&HCI, CPCI-S, CPCI-SSH, ESCI Timespan=All years* |
| # 21 | [24,131](http://apps.webofknowledge.com.proxy.bib.uottawa.ca/summary.do?product=WOS&doc=1&qid=22&SID=3FHSDaBySDxR4AEIsk3&search_mode=GeneralSearch&update_back2search_link_param=yes) | TOPIC: ((("evidence-based" or "evidence based") near/2 (medicine or nursing or practice or pharmacy or "health care" or healthcare)))  *Indexes=SCI-EXPANDED, SSCI, A&HCI, CPCI-S, CPCI-SSH, ESCI Timespan=All years* |
| # 20 | [1,316,688](http://apps.webofknowledge.com.proxy.bib.uottawa.ca/summary.do?product=WOS&doc=1&qid=21&SID=3FHSDaBySDxR4AEIsk3&search_mode=CombineSearches&update_back2search_link_param=yes) | #19 OR #18 OR #17 OR #16 OR #15 OR #14 OR #13 OR #12 OR #11 OR #10 OR #9 OR #8 OR #7 OR #6 OR #5  *Indexes=SCI-EXPANDED, SSCI, A&HCI, CPCI-S, CPCI-SSH, ESCI Timespan=All years* |
| # 19 | [356,502](http://apps.webofknowledge.com.proxy.bib.uottawa.ca/summary.do?product=WOS&doc=1&qid=20&SID=3FHSDaBySDxR4AEIsk3&search_mode=GeneralSearch&update_back2search_link_param=yes) | TOPIC: (("Health Planning" or "health plan$" or "health service$" or "health care" or "health program*" or healthcare))  *Indexes=SCI-EXPANDED, SSCI, A&HCI, CPCI-S, CPCI-SSH, ESCI Timespan=All years* |
| # 18 | [677,355](http://apps.webofknowledge.com.proxy.bib.uottawa.ca/summary.do?product=WOS&doc=1&qid=19&SID=3FHSDaBySDxR4AEIsk3&search_mode=GeneralSearch&update_back2search_link_param=yes) | TOPIC: ((Health Planning or health plan$ or health service$ or health care or health program* or healthcare))  *Indexes=SCI-EXPANDED, SSCI, A&HCI, CPCI-S, CPCI-SSH, ESCI Timespan=All years* |
| # 17 | [26,583](http://apps.webofknowledge.com.proxy.bib.uottawa.ca/summary.do?product=WOS&doc=1&qid=18&SID=3FHSDaBySDxR4AEIsk3&search_mode=GeneralSearch&update_back2search_link_param=yes) | TOPIC: (("government* committee$" or "task force$"))  *Indexes=SCI-EXPANDED, SSCI, A&HCI, CPCI-S, CPCI-SSH, ESCI Timespan=All years* |
| # 16 | [6,774](http://apps.webofknowledge.com.proxy.bib.uottawa.ca/summary.do?product=WOS&doc=1&qid=17&SID=3FHSDaBySDxR4AEIsk3&search_mode=GeneralSearch&update_back2search_link_param=yes) | TOPIC: (((review or advisory) near/1 committee$))  *Indexes=SCI-EXPANDED, SSCI, A&HCI, CPCI-S, CPCI-SSH, ESCI Timespan=All years* |
| # 15 | [69,871](http://apps.webofknowledge.com.proxy.bib.uottawa.ca/summary.do?product=WOS&doc=1&qid=16&SID=3FHSDaBySDxR4AEIsk3&search_mode=GeneralSearch&update_back2search_link_param=yes) | TOPIC: ((Policy near/1 (making or maker or development* or analy*)))  *Indexes=SCI-EXPANDED, SSCI, A&HCI, CPCI-S, CPCI-SSH, ESCI Timespan=All years* |
| # 14 | [6,034](http://apps.webofknowledge.com.proxy.bib.uottawa.ca/summary.do?product=WOS&doc=1&qid=15&SID=3FHSDaBySDxR4AEIsk3&search_mode=GeneralSearch&update_back2search_link_param=yes) | TOPIC: (((contract* near/3 (management or services or tender*))))  *Indexes=SCI-EXPANDED, SSCI, A&HCI, CPCI-S, CPCI-SSH, ESCI Timespan=All years* |
| # 13 | [239,274](http://apps.webofknowledge.com.proxy.bib.uottawa.ca/summary.do?product=WOS&doc=1&qid=14&SID=3FHSDaBySDxR4AEIsk3&search_mode=GeneralSearch&update_back2search_link_param=yes) | TOPIC: (("priorit* setting" or "decision-maker$" or “decision mak*”))  *Indexes=SCI-EXPANDED, SSCI, A&HCI, CPCI-S, CPCI-SSH, ESCI Timespan=All years* |
| # 12 | [8,263](http://apps.webofknowledge.com.proxy.bib.uottawa.ca/summary.do?product=WOS&doc=1&qid=13&SID=3FHSDaBySDxR4AEIsk3&search_mode=GeneralSearch&update_back2search_link_param=yes) | TOPIC: (((((investment OR budget) OR purchas*) OR service) NEAR priority$))  *Indexes=SCI-EXPANDED, SSCI, A&HCI, CPCI-S, CPCI-SSH, ESCI Timespan=All years* |
| # 11 | [49,739](http://apps.webofknowledge.com.proxy.bib.uottawa.ca/summary.do?product=WOS&doc=1&qid=12&SID=3FHSDaBySDxR4AEIsk3&search_mode=GeneralSearch&update_back2search_link_param=yes) | TOPIC: ((service near/2 (development or developer$ or provision or provider$)))  *Indexes=SCI-EXPANDED, SSCI, A&HCI, CPCI-S, CPCI-SSH, ESCI Timespan=All years* |
| # 10 | [148,144](http://apps.webofknowledge.com.proxy.bib.uottawa.ca/summary.do?product=WOS&doc=1&qid=11&SID=3FHSDaBySDxR4AEIsk3&search_mode=GeneralSearch&update_back2search_link_param=yes) | TOPIC: (((commissioning or commissioner$ or purchasing or purchaser$ or procurement or procurer* “budget-holder$” or budgetholder$)))  *Indexes=SCI-EXPANDED, SSCI, A&HCI, CPCI-S, CPCI-SSH, ESCI Timespan=All years* |
| # 9 | [70,425](http://apps.webofknowledge.com.proxy.bib.uottawa.ca/summary.do?product=WOS&doc=1&qid=10&SID=3FHSDaBySDxR4AEIsk3&search_mode=GeneralSearch&update_back2search_link_param=yes) | TOPIC: ((“health insurance” or “medical insurance” or medicare or “managed care”))  *Indexes=SCI-EXPANDED, SSCI, A&HCI, CPCI-S, CPCI-SSH, ESCI Timespan=All years* |
| # 8 | [51,657](http://apps.webofknowledge.com.proxy.bib.uottawa.ca/summary.do?product=WOS&doc=1&qid=9&SID=3FHSDaBySDxR4AEIsk3&search_mode=GeneralSearch&update_back2search_link_param=yes) | TOPIC: ((organi?ational innovation* or organi?ational change* or entrepreneurship))  *Indexes=SCI-EXPANDED, SSCI, A&HCI, CPCI-S, CPCI-SSH, ESCI Timespan=All years* |
| # 7 | [109,533](http://apps.webofknowledge.com.proxy.bib.uottawa.ca/summary.do?product=WOS&doc=1&qid=8&SID=3FHSDaBySDxR4AEIsk3&search_mode=GeneralSearch&update_back2search_link_param=yes) | TOPIC: ((Contract$ or “competitive bid*” or “request for proposal$” or outsourc*))  *Indexes=SCI-EXPANDED, SSCI, A&HCI, CPCI-S, CPCI-SSH, ESCI Timespan=All years* |
| # 6 | [290](http://apps.webofknowledge.com.proxy.bib.uottawa.ca/summary.do?product=WOS&doc=1&qid=7&SID=3FHSDaBySDxR4AEIsk3&search_mode=GeneralSearch&update_back2search_link_param=yes) | TOPIC: (((hospital near/2 purchasing) or "joint purchasing" or "group purchasing"))  *Indexes=SCI-EXPANDED, SSCI, A&HCI, CPCI-S, CPCI-SSH, ESCI Timespan=All years* |
| # 5 | [16,095](http://apps.webofknowledge.com.proxy.bib.uottawa.ca/summary.do?product=WOS&doc=1&qid=6&SID=3FHSDaBySDxR4AEIsk3&search_mode=GeneralSearch&update_back2search_link_param=yes) | TOPIC: (("State medicine" or social* medicine))  *Indexes=SCI-EXPANDED, SSCI, A&HCI, CPCI-S, CPCI-SSH, ESCI Timespan=All years* |
| # 4 | [92,142](http://apps.webofknowledge.com.proxy.bib.uottawa.ca/summary.do?product=WOS&doc=1&qid=5&SID=3FHSDaBySDxR4AEIsk3&search_mode=CombineSearches&update_back2search_link_param=yes) | #3 OR #2 OR #1  *Indexes=SCI-EXPANDED, SSCI, A&HCI, CPCI-S, CPCI-SSH, ESCI Timespan=All years* |
| # 3 | [1,830](http://apps.webofknowledge.com.proxy.bib.uottawa.ca/summary.do?product=WOS&doc=1&qid=4&SID=3FHSDaBySDxR4AEIsk3&search_mode=GeneralSearch&update_back2search_link_param=yes) | TOPIC: ((((gap or gaps) near/7 ((knowledge or research or evidence or trial or result) near/2 practice))))  *Indexes=SCI-EXPANDED, SSCI, A&HCI, CPCI-S, CPCI-SSH, ESCI Timespan=All years* |
| # 2 | [917](http://apps.webofknowledge.com.proxy.bib.uottawa.ca/summary.do?product=WOS&doc=1&qid=3&SID=3FHSDaBySDxR4AEIsk3&search_mode=GeneralSearch&update_back2search_link_param=yes) | TOPIC: (((((systematic near/2 (review* or overview* or synthesis or "literature review*" or "evidence review*")) or "methodolog* review*" or "quantitativ* review*" or "qualitative review*" or "overview or synthes*" or "metasynthes*" or megasynthes*) near/5 (policy or policies or decision*))))  *Indexes=SCI-EXPANDED, SSCI, A&HCI, CPCI-S, CPCI-SSH, ESCI Timespan=All years* |
| # 1 | [89,750](http://apps.webofknowledge.com.proxy.bib.uottawa.ca/summary.do?product=WOS&doc=1&qid=2&SID=3FHSDaBySDxR4AEIsk3&search_mode=GeneralSearch&update_back2search_link_param=yes) | TOPIC: ((("systematic review$" or "methodolog* review$" or "quantitativ* review$" or "qualitative* review$" or "overview$" or "synthes*" or "me?asynthes*") near/5 ("decisionmak*" or "decision-mak*" or "policy-mak*" or "policymak*" or "policy decision*" or "health* polic*" or "health* manag*" or "action*" or "commission*" or "purchas*" or "procur*" or "budget hold*" or "budgethold*" or "service provi*" or "practice" or application or implement* or utili?ation or utili?ing or utili* or disseminat* or summar* or "hospital* decision*" or "treatment plan*" or "patient care" or patientcare or healthcare or "health care" or "clinical decision*" or pathway* or algorithm*)))  *Indexes=SCI-EXPANDED, SSCI, A&HCI, CPCI-S, CPCI-SSH, ESCI Timespan=All years* |

**3. CABI**Run: March 3, 2016

Instructions: Copy and paste the search string. Limit the results by language/date as you see fit. The database doesn’t allow for those limits to be included in the string.

(systematic OR methodolog* OR quantitativ* OR qualitativ* OR review* OR overview* OR synthes* OR metasynthes* OR quantitativ*) AND (( policy OR policies OR decision OR knowledge OR research OR evidence OR trial OR result OR practice OR RCT OR CT) AND gap) AND (diffus* OR disseminat* OR implement* OR adoption OR exchang* OR application OR mobilis* OR mobiliz* OR synthes* OR transfer* OR translat* OR incORpORat* OR uptak* OR utilis* OR utiliz* OR transmission OR integrat* OR democratis* OR democratiz* OR shar* OR broke* OR action* OR collabORat* OR 'cross-profession*' OR intraprofession* OR intra-profession* OR interprofession* OR inter-profession* OR inter-disciplin* OR multi-disciplin* OR multi disciplin* OR multiprofession* OR outsourc* OR subcontract* OR linkage OR exchang* OR KT)

(systematic OR methodolog* OR quantitativ* OR qualitativ* OR review* OR overview* OR synthes* OR metasynthes* OR quantitativ*) AND

​​

((( policy OR policies OR decision OR knowledge OR research OR evidence OR trial OR result OR RCT OR CT) AND practice*) AND gap)

AND

​​

(diffus* OR disseminat* OR implement* OR adoption OR exchang* OR application OR mobilis* OR mobiliz* OR transfer* OR translat* OR incorporat* OR uptak* OR utilis* OR utiliz* OR transmission OR integrat* OR democratis* OR democratiz* OR shar* OR broke* OR action* OR collaborat* OR 'cross-profession*' OR intraprofession* OR intra-profession* OR interprofession* OR inter-profession* OR inter-disciplin* OR multi-disciplin* OR multi disciplin* OR multiprofession* OR outsourc* OR subcontract* OR linkage OR exchang* OR KT)

title:(((systematic OR methodolog* OR quantitativ* OR qualitativ* OR review* OR overview* OR synthes* OR metasynthes* OR quantitativ*)) AND (policy OR policies OR decision OR knowledge OR research OR evidence OR trial OR result OR RCT OR CT) AND (practice AND gap) AND (diffus* OR disseminat* OR implement* OR adoption OR exchang* OR application OR mobilis* OR mobiliz* OR transfer* OR translat* OR incorporat* OR uptak* OR utilis* OR utiliz* OR transmission OR integrat* OR democratis* OR democratiz* OR share* OR broker* OR action* OR collaborat* OR 'cross-profession*' OR intraprofession* OR intra-profession* OR interprofession* OR inter-profession* OR inter-disciplin* OR multi-disciplin* OR multi disciplin* OR multiprofession* OR outsourc* OR subcontract* OR linkage OR exchange OR KT)) OR ab:( ((systematic OR methodolog* OR quantitativ* OR qualitativ* OR review* OR overview* OR synthes* OR metasynthes* OR quantitativ*)) AND (policy OR policies OR decision OR knowledge OR research OR evidence OR trial OR result OR RCT OR CT) AND (practice AND gap) AND (diffus* OR disseminat* OR implement* OR adoption OR exchang* OR application OR mobilis* OR mobiliz* OR transfer* OR translat* OR incorporat* OR uptak* OR utilis* OR utiliz* OR transmission OR integrat* OR democratis* OR democratiz* OR share* OR broker* OR action* OR collaborat* OR 'cross-profession*' OR intraprofession* OR intra-profession* OR interprofession* OR inter-profession* OR inter-disciplin* OR multi-disciplin* OR multi disciplin* OR multiprofession* OR outsourc* OR subcontract* OR linkage OR exchange OR KT))

**3. PROQUEST: Worldwide Political Science Abstracts-9298**

| [Set](http://search.proquest.com.proxy.bib.uottawa.ca/recentsearches.recentsearchtabview.recentsearchesgridview:toggellistorder?site=wpsa&t:ac=RecentSearches) | Search | Databases | Results |
| --- | --- | --- | --- |
| S7 | [S5 and S6](http://search.proquest.com.proxy.bib.uottawa.ca/recentsearches.recentsearchtabview.recentsearchesgridview.scrolledrecentsearchlist.checkdbssearchlink:rerunsearch/1AAE55D679864219PQ/None?site=wpsa&t:ac=RecentSearches) | Worldwide Political Science Abstracts | [66°](http://search.proquest.com.proxy.bib.uottawa.ca/recentsearches.recentsearchtabview.recentsearchesgridview.scrolledrecentsearchlist.checkdbssearchlink_0:rerunsearch/1AAE55D679864219PQ/None?site=wpsa&t:ac=RecentSearches) |
| S6 | [(random* OR controlled OR trial OR “before and after” OR “clinical” OR “time interrupted”)](http://search.proquest.com.proxy.bib.uottawa.ca/recentsearches.recentsearchtabview.recentsearchesgridview.scrolledrecentsearchlist.checkdbssearchlink:rerunsearch/36EF665122924492PQ/None?site=wpsa&t:ac=RecentSearches) | Worldwide Political Science Abstracts | [12,781°](http://search.proquest.com.proxy.bib.uottawa.ca/recentsearches.recentsearchtabview.recentsearchesgridview.scrolledrecentsearchlist.checkdbssearchlink_0:rerunsearch/36EF665122924492PQ/None?site=wpsa&t:ac=RecentSearches) |
| S5 | [S1 OR S2 OR S3 OR S4](http://search.proquest.com.proxy.bib.uottawa.ca/recentsearches.recentsearchtabview.recentsearchesgridview.scrolledrecentsearchlist.checkdbssearchlink:rerunsearch/A01FB1D59E2543D6PQ/None?site=wpsa&t:ac=RecentSearches) | Worldwide Political Science Abstracts | [9,307°](http://search.proquest.com.proxy.bib.uottawa.ca/recentsearches.recentsearchtabview.recentsearchesgridview.scrolledrecentsearchlist.checkdbssearchlink_0:rerunsearch/A01FB1D59E2543D6PQ/None?site=wpsa&t:ac=RecentSearches) |
| S4 | [TI,AB(methodolog* review* OR quantitativ* review* OR qualitative review* OR overview OR synthes* OR metasynthes* OR megasynthes*) AND TI,AB (policy OR policies OR decision*)](http://search.proquest.com.proxy.bib.uottawa.ca/recentsearches.recentsearchtabview.recentsearchesgridview.scrolledrecentsearchlist.checkdbssearchlink:rerunsearch/EA6310944930408CPQ/None?site=wpsa&t:ac=RecentSearches) | Worldwide Political Science Abstracts | [0°](http://search.proquest.com.proxy.bib.uottawa.ca/recentsearches.recentsearchtabview.recentsearchesgridview.scrolledrecentsearchlist.checkdbssearchlink_0:rerunsearch/EA6310944930408CPQ/None?site=wpsa&t:ac=RecentSearches) |
| S3 | [TI,AB(systematic) AND TI,AB(review* OR overview* OR synthes*)](http://search.proquest.com.proxy.bib.uottawa.ca/recentsearches.recentsearchtabview.recentsearchesgridview.scrolledrecentsearchlist.checkdbssearchlink:rerunsearch/EAC1DADDF263462FPQ/None?site=wpsa&t:ac=RecentSearches) | Worldwide Political Science Abstracts | [678°](http://search.proquest.com.proxy.bib.uottawa.ca/recentsearches.recentsearchtabview.recentsearchesgridview.scrolledrecentsearchlist.checkdbssearchlink_0:rerunsearch/EAC1DADDF263462FPQ/None?site=wpsa&t:ac=RecentSearches) |
| S2 | [TI,AB("evidence summary" OR "evidence summaries" OR "policy brief" OR "policy briefs" OR "policy briefing" OR "policy briefings" OR "briefing paper" OR "briefing papers" OR "briefing note" OR "briefing notes" OR "evidence brief" OR "evidence briefs" OR "evidence briefing" OR "evidence briefings" OR "summary of findings" OR "plain language summary" OR "plain language summaries" OR "summarized evidence")](http://search.proquest.com.proxy.bib.uottawa.ca/recentsearches.recentsearchtabview.recentsearchesgridview.scrolledrecentsearchlist.checkdbssearchlink:rerunsearch/4489914F3447460FPQ/None?site=wpsa&t:ac=RecentSearches) | Worldwide Political Science Abstracts | [46°](http://search.proquest.com.proxy.bib.uottawa.ca/recentsearches.recentsearchtabview.recentsearchesgridview.scrolledrecentsearchlist.checkdbssearchlink_0:rerunsearch/4489914F3447460FPQ/None?site=wpsa&t:ac=RecentSearches) |
| S1 | [TI,AB(systematic review* OR methodolog* review* OR quantitativ* review* OR qualitativ* review* OR overview* OR synthes* OR metasynthes* OR megasynthes*) AND TI,AB(decision* OR policy* OR policies OR decision* OR health* OR action* OR commission* OR purchas* OR procur* OR budget* OR service* OR provi* OR practice OR application OR implement* OR utili* OR disseminat* OR summar* OR hospital* OR treatment plan* OR patient* OR clinical OR pathway* OR algorithm*)](http://search.proquest.com.proxy.bib.uottawa.ca/recentsearches.recentsearchtabview.recentsearchesgridview.scrolledrecentsearchlist.checkdbssearchlink:rerunsearch/A628EC9F1AC94181PQ/None?site=wpsa&t:ac=RecentSearches) | Worldwide Political Science Abstracts | [9,038°](http://search.proquest.com.proxy.bib.uottawa.ca/recentsearches.recentsearchtabview.recentsearchesgridview.scrolledrecentsearchlist.checkdbssearchlink_0:rerunsearch/A628EC9F1AC94181PQ/None?site=wpsa&t:ac=RecentSearches) |

**4. WHOLIS –688**

Systematic* OR EBM OR review* OR synthes* OR megasynthes* OR quantitative OR meta* OR literature OR analysis OR summar* OR research* OR result*OR method* OR evidence

AND

Treat* OR path* OR polic* OR gap* OR decis* OR manag* OR procur* OR budget* OR provi* OR patientcare OR purchas* OR commission*OR Diffus* OR util* OR innov* OR mobil* OR appl* OR transfer OR incorpor* OR contract* OR medicare OR service* OR priorit*

**5.PAIS International**

| Search | Databases | Results | Actions |
| --- | --- | --- | --- |
| S7 | [S5 and S6](http://search.proquest.com.proxy.bib.uottawa.ca/recentsearches.recentsearchtabview.recentsearchesgridview.scrolledrecentsearchlist.checkdbssearchlink:rerunsearch/FC9944B0DFD843C7PQ/None?site=pais&t:ac=RecentSearches) | PAIS International | [297°](http://search.proquest.com.proxy.bib.uottawa.ca/recentsearches.recentsearchtabview.recentsearchesgridview.scrolledrecentsearchlist.checkdbssearchlink_0:rerunsearch/FC9944B0DFD843C7PQ/None?site=pais&t:ac=RecentSearches) |
| S6 | [(random* OR controlled OR trial OR “before and after” OR “clinical” OR “time interrupted”)](http://search.proquest.com.proxy.bib.uottawa.ca/recentsearches.recentsearchtabview.recentsearchesgridview.scrolledrecentsearchlist.checkdbssearchlink:rerunsearch/36DD87ACA0CE4388PQ/None?site=pais&t:ac=RecentSearches) | PAIS International | [14,595°](http://search.proquest.com.proxy.bib.uottawa.ca/recentsearches.recentsearchtabview.recentsearchesgridview.scrolledrecentsearchlist.checkdbssearchlink_0:rerunsearch/36DD87ACA0CE4388PQ/None?site=pais&t:ac=RecentSearches) |
| S5 | [S1 OR S2 OR S3 OR S4](http://search.proquest.com.proxy.bib.uottawa.ca/recentsearches.recentsearchtabview.recentsearchesgridview.scrolledrecentsearchlist.checkdbssearchlink:rerunsearch/DD7E7ECD614EAEPQ/None?site=pais&t:ac=RecentSearches) | PAIS International | [9,471°](http://search.proquest.com.proxy.bib.uottawa.ca/recentsearches.recentsearchtabview.recentsearchesgridview.scrolledrecentsearchlist.checkdbssearchlink_0:rerunsearch/DD7E7ECD614EAEPQ/None?site=pais&t:ac=RecentSearches) |
| S4 | [TI,AB(methodolog* review* OR quantitativ* review* OR qualitative review* OR overview OR synthes* OR metasynthes* OR megasynthes*) AND TI,AB (policy OR policies OR decision*)](http://search.proquest.com.proxy.bib.uottawa.ca/recentsearches.recentsearchtabview.recentsearchesgridview.scrolledrecentsearchlist.checkdbssearchlink:rerunsearch/1491C9AC830946DBPQ/None?site=pais&t:ac=RecentSearches) | PAIS International | [0°](http://search.proquest.com.proxy.bib.uottawa.ca/recentsearches.recentsearchtabview.recentsearchesgridview.scrolledrecentsearchlist.checkdbssearchlink_0:rerunsearch/1491C9AC830946DBPQ/None?site=pais&t:ac=RecentSearches) |
| S3 | [TI,AB(systematic) AND TI,AB(review* OR overview* OR synthes*)](http://search.proquest.com.proxy.bib.uottawa.ca/recentsearches.recentsearchtabview.recentsearchesgridview.scrolledrecentsearchlist.checkdbssearchlink:rerunsearch/72E9DE6273C747B9PQ/None?site=pais&t:ac=RecentSearches) | PAIS International | [777°](http://search.proquest.com.proxy.bib.uottawa.ca/recentsearches.recentsearchtabview.recentsearchesgridview.scrolledrecentsearchlist.checkdbssearchlink_0:rerunsearch/72E9DE6273C747B9PQ/None?site=pais&t:ac=RecentSearches) |
| S2 | [TI,AB("evidence summary" OR "evidence summaries" OR "policy brief" OR "policy briefs" OR "policy briefing" OR "policy briefings" OR "briefing paper" OR "briefing papers" OR "briefing note" OR "briefing notes" OR "evidence brief" OR "evidence briefs" OR "evidence briefing" OR "evidence briefings" OR "summary of findings" OR "plain language summary" OR "plain language summaries" OR "summarized evidence")](http://search.proquest.com.proxy.bib.uottawa.ca/recentsearches.recentsearchtabview.recentsearchesgridview.scrolledrecentsearchlist.checkdbssearchlink:rerunsearch/FA2A9E6F8043491DPQ/None?site=pais&t:ac=RecentSearches) | PAIS International | [1,213°](http://search.proquest.com.proxy.bib.uottawa.ca/recentsearches.recentsearchtabview.recentsearchesgridview.scrolledrecentsearchlist.checkdbssearchlink_0:rerunsearch/FA2A9E6F8043491DPQ/None?site=pais&t:ac=RecentSearches) |
| S1 | [TI,AB(systematic review* OR methodolog* review* OR quantitativ* review* OR qualitativ* review* OR overview* OR synthes* OR metasynthes* OR megasynthes*) AND TI,AB(decision* OR policy* OR policies OR decision* OR health* OR action* OR commission* OR purchas* OR procur* OR budget* OR service* OR provi* OR practice OR application OR implement* OR utili* OR disseminat* OR summar* OR hospital* OR treatment plan* OR patient* OR clinical OR pathway* OR algorithm*)](http://search.proquest.com.proxy.bib.uottawa.ca/recentsearches.recentsearchtabview.recentsearchesgridview.scrolledrecentsearchlist.checkdbssearchlink:rerunsearch/1651090E30494A23PQ/None?site=pais&t:ac=RecentSearches) | PAIS International | [8,225°](http://search.proquest.com.proxy.bib.uottawa.ca/recentsearches.recentsearchtabview.recentsearchesgridview.scrolledrecentsearchlist.checkdbssearchlink_0:rerunsearch/1651090E30494A23PQ/None?site=pais&t:ac=RecentSearches) |

**6. Embase Classic+Embase <1947 to 2016 March 22>**

Search Strategy:

--------------------------------------------------------------------------------

1 ((systematic adj2 (review* or overview* or synthesis or literature review* or evidence review*)) or methodolog* review* or quantitativ* review* or qualitative review* or overview or synthes* or metasynthes* or megasynthes*).ti,ab. (1254057)

2 (decision mak* or decision mak* or policy mak* or policy mak* or policy decision* or health* polic* or health* manage* or commission* or purchas* or procur* or budget hold* or budgethold* or service provi* or action* or practice or application or implement* or utili?ation or utili?ing or disseminat* or summar* or hospital* decision* or treatment plan* or patient care or patientcare or healthcare or health care or clinical decision* or pathway* or algorithm*).ti,ab. (4782961)

3 (((systematic adj2 (review* or overview* or synthesis or literature review* or evidence review*)) or methodolog* review* or quantitativ* review* or qualitative review* or overview or synthes* or metasynthes* or megasynthes*) adj5 (decision mak* or decision mak* or policy mak* or policy mak* or policy decision* or health* polic* or health* manage* or commission* or purchas* or procur* or budget hold* or budgethold* or service provi* or action* or practice or application or implement* or utili?ation or utili?ing or disseminat* or summar* or hospital* decision* or treatment plan* or patient care or patientcare or healthcare or health care or clinical decision* or pathway* or algorithm*)).ti,ab. (38202)

4 (((systematic adj2 (review* or overview* or synthesis or literature review* or evidence review*)) or methodolog* review* or quantitativ* review* or qualitative review* or overview or synthes* or metasynthes* or megasynthes*) adj5 (policy or policies or decision*)).ti. (444)

5 ((gap or gaps) adj7 ((knowledge or research or evidence or trial or result) adj2 practice)).ti,ab. (1184)

6 3 or 4 or 5 (39594)

7 Random*.tw. (1075100)

8 Factorial.tw. (26960)

9 (crossover* or cross over*).tw. (81830)

10 placebo.tw. (236848)

11 ((doubl* adj blind*) or (singl* adj blind)).tw. (181725)

12 assign*.tw. (285284)

13 allocat*.tw. (102811)

14 volunteer*.tw. (209615)

15 trial.ti. (203971)

16 crossover-procedure/ or double-blind procedure/ or randomized controlled trial/ or single-blind procedure/ (453472)

17 intervention*.ti. (129607)

18 (intervention* adj6 (clinician* or collaborat* or community or complex or DESIGN* or doctor* or educational or family doctor* or family physician* or family practitioner* or financial or GP or general practice* or hospital* or impact* or improv* or individuali?e* or individuali?ing or interdisciplin* or multicomponent or multi-component or multidisciplin* or multi-disciplin* or multifacet* or multi-facet* or multimodal* or multi-modal* or personali?e* or personali?ing or pharmacies or pharmacist* or pharmacy or physician* or practitioner* or prescrib* or prescription* or primary care or professional* or provider* or regulatory or regulatory or tailor* or target* or team* or usual care)).ab. (189458)

19 (collaborativ* or collaboration* or tailored or personali?ed).ti,ab. (189368)

20 (exp *hospital/ or *hospitalization/ or exp *patient/ or exp *nurse/ or exp *nursing/) and (study.ti. or *evaluation/) (67150)

21 demonstration project*.ti,ab. (2638)

22 (pre-post or "pre test*" or pretest* or posttest* or "post test*" or (pre adj5 post)).ti,ab. (128204)

23 (pre-workshop or post-workshop or (before adj3 workshop) or (after adj3 workshop)).ti,ab. (1141)

24 ((study adj3 aim?) or "our study").ab. (942277)

25 (before adj10 (after or during)).ti,ab. (550438)

26 ("quasi-experiment$" or quasiexperiment$ or "quasi random$" or quasirandom$ or "quasi control$" or quasicontrol$ or ((quasi$ or experimental) adj3 (method$ or study or trial or design$))).ti,ab. (145698)

27 ("time series" adj2 interrupt*).ti,ab,hw. (1650)

28 (time points adj3 (over or multiple or three or four or five or six or seven or eight or nine or ten or eleven or twelve or month* or hour? or day? or "more than")).ab. (16552)

29 pilot.ti. (63098)

30 *experimental design/ or *pilot study/ or quasi experimental study/ (12430)

31 clinical trial/ or randomized controlled trial/ or controlled clinical trial/ (1006393)

32 multicenter study/ (133719)

33 (multicentre or multicenter or multi-centre or multi-center).ti. (51298)

34 random*.ti,ab. or controlled.ti. (1137856)

35 (control adj3 (area or cohort? or compar? or condition or group? or intervention? or participant? or study)).ab. (633237)

36 *drug therapy/ and irrational.ti,ab. (39)

37 (rational adj4 (drug therapy or "drug use" or prescribing)).ti,ab. (1568)

38 (rational or irrational).ti. and drug therapy.hw. (578)

39 ((promote or prefer) adj5 generic).ti,ab. (98)

40 prescribing habits.ti,ab. (988)

41 ((physician* or doctor* or nurse*) adj4 compliance).ti,ab. (2242)

42 promoting.ti. and (health* or care or education or nurse* or nursing or patient* or hospital*).ti,hw. (8646)

43 (promoting and (doctor* or physician* or pharmacist*)).ti. (172)

44 (fund-hold* or fundhold* or capitation or capitated or copay* or co-pay*).ti,ab. (6442)

45 (impact or effect* or change? or changing).ti. (2717843)

46 (communit* or team* or interdisciplinar* or multidisciplinar*).ti,ab. (705692)

47 (implementation or implementing).ti. and (care or healthcare).ti,hw. (17617)

48 ((effect? or effectiveness or chang* or improv* or impact) adj3 practice).ti,ab. (34899)

49 (Improv* adj3 (diagnosis or treatment? or prescribing)).ti,ab. (109203)

50 ((evidence or evidence-based) adj4 intervention).ti,ab. (4210)

51 evidence driven.ti,ab. (134)

52 practice based.ti,ab. (5417)

53 (improv* adj3 (decision* or implement* or health care or healthcare or initiative? or management or multifacet* or multi-facet* or multi-component or practi?e? or practitioner? or prescrib* or prescription? or professional? or program? or programme? or provider?)).ti. (13425)

54 (improv* adj2 (patient-care or family practice or ((family or general) adj2 (practi?e or practitioner? or doctor?)) or primary care)).ab. (8824)

55 recommended practice?.ti,ab. (1026)

56 ((information or evidence) adj2 uptake).ti,ab. (664)

57 ((knowledge adj2 (application or broke* or creation or diffus* or disseminat* or exchang* or implement* or management or mobili* or translat* or transfer* or uptake or utili*)) or (evidence* adj2 (exchang* or translat* or transfer*))).ti,ab. (14737)

58 (KT adj2 (application or broke* or diffus* or disseminat* or decision* or exchang* or implement* or intervent* or mobili* or plan* or policy or policies or strateg* or translat* or transfer* or uptake or utili*)).ti,ab. (416)

59 ((research or knowledge or innovation* or evidence) adj5 (change* or changing or improv* or promot* or influenc* or impact* or disinvest* or discontinu* or reject* or abandon* or ceas* or restrict* or disincentiv* or stop*)).ti,ab. (152806)

60 "linkage and exchange".ti,ab. (18)

61 (push activit* or pull activit*).ti,ab. (10)

62 ((computer-tailored or individuali?ing or individuali?ed or personali?e? or personali?ing or tailor*) adj2 (feedback or intervention? or information or plan?)).ti,ab. (10153)

63 ((conventional or evidence-based or pattern or regular or routine or standard or traditional or usual) adj2 (care or healthcare or patient care or practice)).ti,ab. (116871)

64 (collaborative? or interdisciplin* or inter-disciplin* or multidisciplin* or multi-disciplin* or team? or team-based or skill-mix).ti. (56400)

65 ((collaborative or multidisciplinary or interdisciplinary) adj2 (care or healthcare or patient care or team?)).ab. (31303)

66 (skill? adj2 (mix or mixes)).ti,ab. (896)

67 (doctor-driven or doctor-led or GP-LED or nurse-led or nurse-driven or pharmacist-led or pharmacist-driven or physician-led or physician-driven).ti,ab. (5552)

68 physician directed.ti,ab. (414)

69 (booklet* or leaflet* or pamphlet* or "written information").ti. or ((BOOKLET? or leaflet* or pamphlet* or "written information") adj5 (intervention? or care or healthcare or physician? or practitioner? or provider?)).ab. (5351)

70 (academic detailing or e-detailing or (opinion? adj2 leader?)).ti,ab. (1844)

71 ("audit and feedback" or ((physician? or doctor? or practitioner? or nurse? or provider?) adj feedback)).ti,ab. (1247)

72 reminder?.ti. (1979)

73 (reminder? adj2 (clinician? or physician? or practitioner? or nurse? or doctor? or provider?)).ab. (468)

74 ((clinician? or physician?) adj2 (prompt or prompts or prompting)).ti,ab. (954)

75 ((doctor? or nurse? or pharmacist? or physician? or practitioner?) adj2 behavio?r?).ti,ab. (3873)

76 (nurse? adj4 substitut*).ti,ab. (144)

77 (practice pattern? or ((change? or changing) adj2 practice)).ti,ab. (17288)

78 (nurse-practitioner? or physician? assistant?).ti. (5915)

79 ((doctor? or nurse? or pharmacist? or physician?) adj2 role?).ab. (7934)

80 ((nurse? or physician? or pharmacist? or provider?) adj2 initiative?).ti,ab. (548)

81 (virtual reality or VR Training or VR simulat* or (simulat* adj2 skill?)).ti,ab. (7401)

82 (blog* or wiki* or PDA or "palm pilot" or podcast* or blackberr* or Twitter or tweet or tweeting or facebook or social networking or social marketing or youtube).ti,ab. or blogging/ (101294)

83 (health 20 or healthcare 20 or health care 20 or web 20).ti,ab. (671)

84 ((research or knowledge or innovation* or evidence or information or policy) adj5 (brief* or summar* or structured summar* synops* or overview* or bulletin* or synthes* or map or mapping or maps or framing* or product* or package* or alert* or comment* or strateg* or algorithm* or decision-aid* or decisionaid*)).ti,ab. (145362)

85 (((individuali* or integrated) adj2 (care or healthcare or medical care)) or patient-centred or patient-centered or patient-control*).ti,ab. (32415)

86 quality improvement.ti,ab. (27828)

87 *patient satisfaction/ (17293)

88 (algorithm? and (care or healthcare or patient?)).ti,hw. (45023)

89 ((continuing adj2 (education* or training)) or (skill? adj (education or training))).ti,ab. (27705)

90 (reminder? adj2 (clinician? or physician? or practitioner? or nurse? or doctor? or provider?)).ab. (468)

91 (Referral? adj3 (early or increase? or primary care or specialist? or general practitioner? or optimi?e? or optimal or reduce? or reducing)).ab. (9966)

92 referral?.ti. (15646)

93 (specialist? and (primary care or primary healthcare or GP or general practitioner? or family doctor)).ti. (817)

94 (specialist? adj3 (primary care or primary healthcare or GP or general practitioner? or family doctor)).ab. (3911)

95 *reminder system/ (879)

96 patient referral/ (72676)

97 or/7-96 (7508913)

98 (animal model? or animal experiment? or animal study? or animal trial? or canine or feline or bovine or cow or cows or mice or dog? or cat or cats or rabbit? or rat or rats or veterinar*).ti. or (animal or veterinary).hw. (5336801)

99 (editorial or letter or note or "review" or trade or survey).pt. (4539652)

100 meta-analysis/ or systematic review/ or "literature review".ti. or "systematic review".ti. or (meta-analy* or metaanalyt*).ti. (216562)

101 98 or 99 or 100 (9657204)

102 97 not 101 (5303196)

103 6 and 102 (5211)

104 limit 103 to yr="1992 -Current" (4537)

**7. PsycINFO search strategy**

3. (((systematic adj2 (review* or overview* or synthesis or literature review* or evidence review*)) or methodolog* review* or quantitativ* review* or qualitative review* or overview or synthes* or metasynthes* or megasynthes*) adj5 (decision mak* or decision mak* or policy mak* or policy mak* or policy decision* or health* polic* or health* manage* or commission* or purchas* or procur* or budget hold* or budgethold* or service provi* or action* or practice or application or implement* or utili?ation or utili?ing or disseminat* or summar* or hospital* decision* or treatment plan* or patient care or patientcare or healthcare or health care or clinical decision* or pathway* or algorithm*)).ti,ab.

4. (((systematic adj2 (review* or overview* or synthesis or literature review* or evidence review*)) or methodolog* review* or quantitativ* review* or qualitative review* or overview or synthes* or metasynthes* or megasynthes*) adj5 (policy or policies or decision*)).ti.

5. ((gap or gaps) adj7 ((knowledge or research or evidence or trial or result) adj2 practice)).ti,ab.

6. 3 or 4 or 5

7. placebo.tw.

8. random*.tw.

9. ((doubl* adj blind*) or (singl* adj blind)).tw.

10. assign*.tw.

11. allocat*.tw.

12. volunteer*.tw.

13. trial.ti.

14. clinical trials/

15. intervention/

16. intervention*.ti.

17. (intervention* adj6 (clinician* or collaborat* or community or complex or DESIGN* or doctor* or educational or family doctor* or family physician* or family practitioner* or financial or GP or general practice* or hospital* or impact* or improv* or individuali?e* or individuali?ing or interdisciplin* or multicomponent or multi-component or multidisciplin* or multi-disciplin* or multifacet* or multi-facet* or multimodal* or multi-modal* or personali?e* or personali?ing or pharmacies or pharmacist* or pharmacy or physician* or practitioner* or prescrib* or prescription* or primary care or professional* or provider* or regulatory or regulatory or tailor* or target* or team* or usual care)).ab.

18. (collaborativ* or collaboration* or tailored or personali?ed).ti,ab.

19. (hospital/ or Hospitalization/ or exp Patients/ or exp Nurses/ or Nursing/) and (study.ti. or evaluation/ or treatment effectiveness evaluation/)

20. demonstration project*.ti,ab.

21. (pre-post or "pre test*" or pretest* or posttest* or "post test*" or (pre adj5 post)).ti,ab.

22. (pre-workshop or post-workshop or (before adj3 workshop) or (after adj3 workshop)).ti,ab.

23. ((study adj3 aim?) or "our study").ab.

24. (before adj10 (after or during)).ti,ab.

25. ("quasi-experiment$" or quasiexperiment$ or "quasi random$" or quasirandom$ or "quasi control$" or quasicontrol$ or ((quasi$ or experimental) adj3 (method$ or study or trial or design$))).ti,ab.

26. ("time series" adj2 interrupt*).ti,ab,hw.

27. (time points adj3 (over or multiple or three or four or five or six or seven or eight or nine or ten or eleven or twelve or month* or hour? or day? or "more than")).ab.

28. pilot.ti.

29. (multicentre or multicenter or multi-centre or multi-center).ti.

30. random*.ti,ab. or controlled.ti.

31. (control adj3 (area or cohort? or compar? or condition or group? or intervention? or participant? or study)).ab.

32. drug therapy/ and irrational.ti,ab.

33. (rational adj4 (drug therapy or "drug use" or prescribing)).ti,ab.

34. (rational or irrational).ti. and drug therapy.hw.

35. ((promote or prefer) adj5 generic).ti,ab.

36. prescribing habits.ti,ab.

37. ((physician* or doctor* or nurse*) adj4 compliance).ti,ab.

38. promoting.ti. and (health* or care or education or nurse* or nursing or patient* or hospital*).ti,hw.

39. (promoting and (doctor* or physician* or pharmacist*)).ti.

40. (fund-hold* or fundhold* or capitation or capitated or copay* or co-pay*).ti,ab.

41. (impact or effect* or change? or changing).ti.

42. (communit* or team* or interdisciplinar* or multidisciplinar*).ti,ab.

43. (implementation or implementing).ti. and (care or healthcare).ti,hw.

44. ((effect? or effectiveness or chang* or improv* or impact) adj3 practice).ti,ab.

45. (Improv* adj3 (diagnosis or treatment? or prescribing)).ti,ab.

46. ((evidence or evidence-based) adj4 intervention).ti,ab.

47. evidence driven.ti,ab.

48. practice based.ti,ab.

49. (improv* adj3 (decision* or implement* or health care or healthcare or initiative? or management or multifacet* or multi-facet* or multi-component or practi?e? or practitioner? or prescrib* or prescription? or professional? or program? or programme? or provider?)).ti.

50. (improv* adj2 (patient-care or family practice or ((family or general) adj2 (practi?e or practitioner? or doctor?)) or primary care)).ab.

51. recommended practice?.ti,ab.

52. ((information or evidence) adj2 uptake).ti,ab.

53. ((knowledge adj2 (application or broke* or creation or diffus* or disseminat* or exchang* or implement* or management or mobili* or translat* or transfer* or uptake or utili*)) or (evidence* adj2 (exchang* or translat* or transfer*))).ti,ab.

54. (KT adj2 (application or broke* or diffus* or disseminat* or decision* or exchang* or implement* or intervent* or mobili* or plan* or policy or policies or strateg* or translat* or transfer* or uptake or utili*)).ti,ab.

55. ((research or knowledge or innovation* or evidence) adj5 (change* or changing or improv* or promot* or influenc* or impact* or disinvest* or discontinu* or reject* or abandon* or ceas* or restrict* or disincentiv* or stop*)).ti,ab.

56. "linkage and exchange".ti,ab.

57. (push activit* or pull activit*).ti,ab.

58. ((computer-tailored or individuali?ing or individuali?ed or personali?e? or personali?ing or tailor*) adj2 (feedback or intervention? or information or plan?)).ti,ab.

59. ((conventional or evidence-based or pattern or regular or routine or standard or traditional or usual) adj2 (care or healthcare or patient care or practice)).ti,ab.

60. (collaborative? or interdisciplin* or inter-disciplin* or multidisciplin* or multi-disciplin* or team? or team-based or skill-mix).ti.

61. ((collaborative or multidisciplinary or interdisciplinary) adj2 (care or healthcare or patient care or team?)).ab.

62. (skill? adj2 (mix or mixes)).ti,ab.

63. (doctor-driven or doctor-led or GP-LED or nurse-led or nurse-driven or pharmacist-led or pharmacist-driven or physician-led or physician-driven).ti,ab.

64. physician directed.ti,ab.

65. (booklet* or leaflet* or pamphlet* or "written information").ti. or ((BOOKLET? or leaflet* or pamphlet* or "written information") adj5 (intervention? or care or healthcare or physician? or practitioner? or provider?)).ab.

66. (academic detailing or e-detailing or (opinion? adj2 leader?)).ti,ab.

67. ("audit and feedback" or ((physician? or doctor? or practitioner? or nurse? or provider?) adj feedback)).ti,ab.

68. reminder?.ti.

69. (reminder? adj2 (clinician? or physician? or practitioner? or nurse? or doctor? or provider?)).ab.

70. ((clinician? or physician?) adj2 (prompt or prompts or prompting)).ti,ab.

71. ((doctor? or nurse? or pharmacist? or physician? or practitioner?) adj2 behavio?r?).ti,ab.

72. (nurse? adj4 substitut*).ti,ab.

73. (practice pattern? or ((change? or changing) adj2 practice)).ti,ab.

74. (nurse-practitioner? or physician? assistant?).ti.

75. ((doctor? or nurse? or pharmacist? or physician?) adj2 role?).ab.

76. ((nurse? or physician? or pharmacist? or provider?) adj2 initiative?).ti,ab.

77. (virtual reality or VR Training or VR simulat* or (simulat* adj2 skill?)).ti,ab.

78. (blog* or wiki* or PDA or "palm pilot" or podcast* or blackberr* or Twitter or tweet or tweeting or facebook or social networking or social marketing or youtube).ti,ab. or blogging/

79. (health 20 or healthcare 20 or health care 20 or web 20).ti,ab.

80. ((research or knowledge or innovation* or evidence or information or policy) adj5 (brief* or summar* or structured summar* synops* or overview* or bulletin* or synthes* or map or mapping or maps or framing* or product* or package* or alert* or comment* or strateg* or algorithm* or decision-aid* or decisionaid*)).ti,ab.

81. (((individuali* or integrated) adj2 (care or healthcare or medical care)) or patient-centred or patient-centered or patient-control*).ti,ab.

82. quality improvement.ti,ab.

83. client satisfaction/

84. (algorithm? and (care or healthcare or patient?)).ti,hw.

85. ((continuing adj2 (education* or training)) or (skill? adj (education or training))).ti,ab.

86. (reminder? adj2 (clinician? or physician? or practitioner? or nurse? or doctor? or provider?)).ab.

87. (Referral? adj3 (early or increase? or primary care or specialist? or general practitioner? or optimi?e? or optimal or reduce? or reducing)).ab.

88. referral?.ti.

89. (specialist? and (primary care or primary healthcare or GP or general practitioner? or family doctor)).ti.

90. (specialist? adj3 (primary care or primary healthcare or GP or general practitioner? or family doctor)).ab.

91. or/7-90

92. exp animals/ or animal models/

93. ("literature review" or "systematic review" or (meta-analy* or metaanalyt*)).ti.

94. 92 or 93

95. 91 not 94

96. 6 and 95

**8. CINAHL search strategy**

| # | Query | Results |
| --- | --- | --- |
| S55 | S33 AND S54 | 656 |
| S54 | S46 AND S53 | 80,397 |
| S53 | S47 OR S48 OR S49 OR S50 OR S51 OR S52 | 167,143 |
| S52 | TI ( “control* N1 clinical” or “control* N1 group*” or “control* N1 trial*” or “control* N1 study” or “control* N1 studies” or “control* N1 design*” or “control* N1 method*” ) or AB ( “control* N1 clinical” or “control* N1 group*” or “control* N1 trial*” or “control* N1 study” or “control* N1 studies” or “control* N1 design*” or “control* N1 method*” ) | 1 |
| S51 | TI controlled or AB controlled | 73,626 |
| S50 | TI random* or AB random* | 126,367 |
| S49 | TI ( “clinical study” or “clinical studies” ) or AB ( “clinical study” or “clinical studies” ) | 7,891 |
| S48 | (MM "Clinical Trials+") | 9,265 |
| S47 | TI ( (multicent* n2 design*) or (multicent* n2 study) or (multicent* n2 studies) or (multicent* n2 trial*) ) or AB ( (multicent* n2 design*) or (multicent* n2 study) or (multicent* n2 studies) or (multicent* n2 trial*) ) | 9,991 |
| S46 | S34 OR S35 OR S36 OR S37 OR S38 OR S39 OR S40 OR S41 OR S42 OR S43 OR S44 OR S45 | 245,886 |
| S45 | TI ( (time points n3 over) or (time points n3 multiple) or (time points n3 three) or (time points n3 four) or (time points n3 five) or (time points n3 six) or (time points n3 seven) or (time points n3 eight) or (time points n3 nine) or (time points n3 ten) or (time points n3 eleven) or (time points n3 twelve) or (time points n3 month*) or (time points n3 hour*) or (time points n3 day*) or (time points n3 "more than") ) or AB ( (time points n3 over) or (time points n3 multiple) or (time points n3 three) or (time points n3 four) or (time points n3 five) or (time points n3 six) or (time points n3 seven) or (time points n3 eight) or (time points n3 nine) or (time points n3 ten) or (time points n3 eleven) or (time points n3 twelve) or (time points n3 month*) or (time points n3 hour*) or (time points n3 day*) or (time points n3 "more than") ) | 3,123 |
| S44 | TI ( (control w3 area) or (control w3 cohort*) or (control w3 compar*) or (control w3 condition) or (control w3 group*) or (control w3 intervention*) or (control w3 participant*) or (control w3 study) ) or AB ( (control w3 area) or (control w3 cohort*) or (control w3 compar*) or (control w3 condition) or (control w3 group*) or (control w3 intervention*) or (control w3 participant*) or (control w3 study) ) | 53,510 |
| S43 | TI (random* OR controlled) | 41,875 |
| S42 | TI ( collaborativ* or collaboration* or tailored or personalised or personalized ) or AB ( collaborativ* or collaboration* or tailored or personalised or personalized ) | 43,667 |
| S41 | TI pilot | 14,161 |
| S40 | (MH "Pilot Studies") | 34,652 |
| S39 | AB "before-and-after" | 19,657 |
| S38 | AB time series | 2,090 |
| S37 | TI time series | 326 |
| S36 | AB ( before* n10 during or before n10 after ) or AU ( before* n10 during or before n10 after ) | 36,834 |
| S35 | TI ( (time point*) or (period* n4 interrupted) or (period* n4 multiple) or (period* n4 time) or (period* n4 various) or (period* n4 varying) or (period* n4 week*) or (period* n4 month*) or (period* n4 year*) ) or AB ( (time point*) or (period* n4 interrupted) or (period* n4 multiple) or (period* n4 time) or (period* n4 various) or (period* n4 varying) or (period* n4 week*) or (period* n4 month*) or (period* n4 year*) ) | 55,428 |
| S34 | TI ( ( quasi-experiment* or quasiexperiment* or quasi-random* or quasirandom* or quasi control* or quasicontrol* or quasi* W3 method* or quasi* W3 study or quasi* W3 studies or quasi* W3 trial or quasi* W3 design* or experimental W3 method* or experimental W3 study or experimental W3 studies or experimental W3 trial or experimental W3 design* ) ) or AB ( ( quasi-experiment* or quasiexperiment* or quasi-random* or quasirandom* or quasi control* or quasicontrol* or quasi* W3 method* or quasi* W3 study or quasi* W3 studies or quasi* W3 trial or quasi* W3 design* or experimental W3 method* or experimental W3 study or experimental W3 studies or experimental W3 trial or experimental W3 design* ) ) | 13,693 |
| S33 | S1 or S2 or S3 or S4 or S5 or S6 or S7 or S8 or S9 or S10 or S11 or S12 or S13 or S14 or S15 or S16 or S17 or S18 or S19 or S20 or S21 or S22 or S23 or S24 or S25 or S26 or S27 or S28 or S29 or S30 or S31 or S32 | 8,076 |
| S32 | ""( gap n7 knowledge n2 practice or gap n7 research n2 practice or gap n7 evidence n2 practice or gap n7 trial n2 practice or gap n7 result n2 practice ) or ( gaps n7 knowledge n2 practice or gaps n7 research n2 practice or gaps n7 evidence n2 practice or gaps n7 trial n2 practice or gaps n7 result n2 practice )"" | 757 |
| S31 | TI ( methodolog* review* n5 policy or quantitative review*n5 policy or qualitative review* n5 policy or overview* n5 policy or synthes* n5 policy or megasynthes* n5 policy or metasynthes* n5 policyor methodolog* review* n5 policies or quantitative review*n5 policies or qualitative review* n5 policies or overview* n5 policies or synthes* n5 policies or megasynthes* n5 policies or metasynthes* n5 policies ) or TI ( methodolog* review* n5 decision* or quantitative review*n5 decision* or qualitative review* n5 decision* or overview* n5 decision* or synthes* n5 decision* or megasynthes* n5 decision* or metasynthes* n5 decision* )TI ( methodolog* review* n5 policy or quantitative review*n5 policy or qualitative review* n5 policy or overview* n5 policy or synthes* n5 policy or megasynthes* n5 policy or metasynthes* n5 policyor methodolog* review* n5 policies or quantitative review*n5 policies or qualitative review* n5 policies or overview* n5 policies or synthes* n5 policies or megasynthes* n5 policies or metasynthes* n5 policies ) or TI ( methodolog* review* n5 decision* or quantitative review*n5 decision* or qualitative *n5 decision* | 83 |
| S30 | TI ( systematic n2 review* n5 decision* or systematic n2 overview* n5 decision* or systematic n2 synthesis n5 decision* or systematic n2 literature review* n5 decision* or systematic n2 evidence review* ) or TI ( systematic n2 review* n5 policy or systematic n2 overview* n5 policy or systematic n2 synthesis n5 policy or systematic n2 literature review* n5 policy or systematic n2 evidence review* or systematic n2 review* n5 policies or systematic n2 overview* n5 policies or systematic n2 synthesis n5 policies or systematic n2 literature review* n5 policies systematic n2 evidence review* )TI ( systematic n2 review* n5 decision* or systematic n2 overview* n5 decision* or systematic n2 synthesis n5 decision* or systematic n2 literature review* n5 decision* or systematic n2 evidence review* ) or TI ( systematic n2 review* n5 policy or systematic n2 overview* n5 policy or systematic n2 synthesis n5 policy or systematic n2 literature review* n5 policy or systematic n2 evidence review* or systematic n2 review* n5 policies or systematic n2 overview* n5 policies or systematic n2 synthesis n5 policies | 2,341 |
| S29 | (methodolog* review* n5 pathway* or quantitative review*n5 pathway* or qualitative review* n5 pathway* or overview* n5 pathway* or synthes* n5 pathway* or megasynthes* n5 pathway* or metasynthes* n5 pathway*or methodolog* review* n5 algorithm* or quantitative review*n5 algorithm* or qualitative review* n5 algorithm* or overview* n5 algorithm* or synthes* n5 algorithm* or megasynthes* n5 algorithm* or metasynthes* n5 algorithm*) | 197 |
| S28 | (methodolog* review* n5 health care or quantitative review*n5 health care or qualitative review* n5 health care or overview* n5 health care or synthes* n5 health care or megasynthes* n5 health care or metasynthes* n5 health care or methodolog* review* n5 healthcare or quantitative review*n5 healthcare or qualitative review* n5 healthcare or overview* n5 healthcare or synthes* n5 healthcare or metasynthes* n5 healthcare or megasynthes* n5 healthcare) | 508 |
| S27 | ""methodolog* review* n5 patient care or quantitative review*n5 patient care or qualitative review* n5 patient care or overview* n5 patient care or synthes* n5 patient care or megasynthes* n5 patient care or metasynthes* n5 patient care or methodolog* review* n5 patient care or quantitative review*n5 patient care or qualitative review* n5 patient care or overview* n5 patient care or synthes* n5 patient care or megasynthes* n5 patient care or metasynthes* n5 patient care"" | 206 |
| S26 | (methodolog* review* n5 treatment plan* or quantitative review*n5 treatment plan* or qualitative review* n5 treatment plan* or overview* n5 treatment plan* or synthes* n5 treatment plan* or megasynthes* n5 treatment plan* or metasynthes* n5 treatment plan* or methodolog* review* n5 clinical decision* or quantitative review*n5 clinical decision* or qualitative review* n5 hospital* decision* or overview* n5 clinical decision* or synthes* n5 clinical decision* or megasynthes* n5 clinical decision* or metasynthes* n5 clinical decision*methodolog* review* n5 treatment plan* or quantitative review*n5 treatment plan* or qualitative review* n5 treatment plan* or overview* n5 treatment plan* or synthes* n5 treatment plan* or megasynthes* n5 treatment plan* or metasynthes* n5 treatment plan* or methodolog* review* n5 clinical decision* or quantitative review*n5 clinical decision* or qualitative review* n5 hospital* decision* or overview* n5 clinical decision* or synthes* n5 clinical decision* or megasynthes* n5 clinical decision* or metasynthes* n5 clinical decision*methodolog* review* n5 treatment plan*) | 49 |
| S25 | (methodolog* review* n5 summar* or quantitative review*n5 summar* or qualitative review* n5 summar* or overview* n5 summar* or synthes* n5 summar* or megasynthes* n5 summar* or metasynthes* n5 summar* or methodolog* review* n5 hospital* decision* or quantitative review*n5 hospital* decision* or qualitative review* n5 hospital* decision* or overview* n5 hospital* decision* or synthes* n5 hospital* decision* or megasynthes* n5 hospital* decision* or metasynthes* n5 hospital* decision*) | 541 |
| S24 | (methodolog* review* n5 utili* or quantitative review*n5 utili* or qualitative review* n5 utili* or overview* n5 utili* or synthes* n5 utili* or megasynthes* n5 utili* or megasynthes* n5 utili* or methodolog* review* n5 disseminat* or quantitative review*n5 disseminat* or qualitative review* n5 disseminat* or overview* n5 disseminat* or synthes* n5 disseminat* or megasynthes* n5 disseminat* or metasynthes* n5 disseminat*) | 221 |
| S23 | (methodolog* review* n5 application or quantitative review*n5 application or qualitative review* n5 application or overview* n5 application or synthes* n5 application or megasynthes* n5 application or metasynthes* n5 application or methodolog* review* n5 implement* or quantitative review*n5 implement* or qualitative review* n5 implement* or overview* n5 implement* or synthes* n5 implement* or megasynthes* n5 implement* or metasynthes* n5 implement*) | 425 |
| S22 | (methodolog* review* n5 action* or quantitative review*n5 action* or qualitative review* n5 action* or overview* n5 action* or synthes* n5 action* or megasynthes* n5 action* or metasynthes* n5 action* or methodolog* review* n5 practice or quantitative review*n5 practice or qualitative review* n5 practice or overview* n5 practice or synthes* n5 practice or megasynthes* n5 practice or metasynthes* n5 practice) | 995 |
| S21 | ""methodolog* review* n5 budgethold* or quantitative review*n5 budgethold* or qualitative review* n5 budgethold* or overview* n5 budgethold* or synthes* n5 budgethold* or megasynthes* n5 budgethold* or metasynthes* n5 budgethold* or methodolog* review* n5 budget hold* or quantitative review*n5 budget hold* or qualitative review* n5 budget hold* or overview* n5 budget hold* or synthes* n5 budget hold* or megasynthes* n5 budget hold* or metasynthes* n5 budget hold*"" | 0 |
| S20 | (methodolog* review* n5 budgethold* or quantitative review*n5 budgethold* or qualitative review* n5 budgethold* or overview* n5 budgethold* or synthes* n5 budgethold* or megasynthes* n5 budgethold* or metasynthes* n5 budgethold* or methodolog* review* n5 budget hold* or quantitative review*n5 budget hold* or qualitative review* n5 budget hold* or overview* n5 budget hold* or synthes* n5 budget hold* or megasynthes* n5 budget hold* or metasynthes* n5 budget hold*) | 0 |
| S19 | ""methodolog* review* n5 procur* or quantitative review*n5 procur* or qualitative review* n5 procur* or overview* n5 procur* or synthes* n5 procur* or megasynthes* n5 procur* or metasynthes* n5 procur* or methodolog* review* n5 service provi* or quantitative review*n5 service provi* or qualitative review* n5 service provi* or overview* n5 service provi* or synthes* n5 service provi* or megasynthes* n5 service provi* or metasynthes* n5 service provi*"" | 101 |
| S18 | ""methodolog* review* n5 commission* or quantitative review*n5 commission* or qualitative review* n5 commission* or overview* n5 commission* or synthes* n5 commission* or megasynthes* n5 commission* or metasynthes* n5 commission* or methodolog* review* n5 purchas* or quantitative review*n5 purchas* or qualitative review* n5 purchas* or overview* n5 purchas* or synthes* n5 purchas* or megasynthes* n5 purchas* or metasynthes* n5 purchas*"" | 27 |
| S17 | ""methodolog* review* n5 policy decision* or quantitative review*n5 policy decision* or qualitative review* n5 policy decision* or overview* n5 policy decision* or synthes* n5 policy decision* or megasynthes* n5 policy decision* or metasynthes* n5 health manage* or methodolog* review* n5 health manage* or quantitative review*n5 health manage* or qualitative review* n5 health manage* or overview* n5 health manage* or synthes* n5 health manage* or megasynthes* n5 health manage* or metasynthes* n5 health manage*methodolog* review* n5 policy decision* or quantitative review*n5 policy decision* or qualitative review* n5 policy decision* or overview* n5 policy decision* or synthes* n5 policy decision* or megasynthes* n5 policy decision* or metasynthes* n5 health manage* or methodolog* review* n5 health manage* or quantitative review*n5 health manage* or qualitative review* n5 health manage* or overview* n5 health manage* or synthes* n5 health manage* or megasynthes* n5 health manage* or metasynthes* n5 health manage*methodolog* review* n5 policy decision*"" | 36 |
| S16 | ""methodolog* review* n5 policy making or quantitative review*n5 policy making or qualitative review* n5 policy making or overview* n5 policy making or synthes* n5 policy making or megasynthes* n5 policy making or metasynthes* n5 policy making or methodolog* review* n5 policy making or quantitative review*n5 policy making or qualitative review* n5 policy making or overview* n5 policy making or synthes* n5 policy making or megasynthes* n5 policy making or megasynthes* n5 policy making"" | 6 |
| S15 | ""methodolog* review* n5 decision making or quantitative review*n5 decision making or qualitative review* n5 decision making or overview* n5 decision making or synthes* n5 decision making or megasynthes* n5 decision making or methodolog* review* n5 decision-making or quantitative review*n5 decision-making or qualitative review* n5 decision-making or overview* n5 decision-making or synthes* n5 decision-making or megasynthes* n5 decision-making or megasynthes* n5 decision-making"" | 92 |
| S14 | ""systematic n2 review* n5 pathway* or systematic n2 overview*n5 pathway* or systematic n2 synthesis n5 pathway* or systematic n2 syntheses n5 pathway* or systematic n2 literature review* n5 pathway* or systematic n2 evidence review* n5 pathway* or systematic n2 review* n5 algorithm* or systematic n2 overview*n5 algorithm* or systematic n2 synthesis n5 algorithm* or systematic n2 syntheses n5 algorithm* or systematic n2 literature review* n5 algorithm* or systematic n2 evidence review* n5 algorithm*systematic n2 review* n5 pathway* or systematic n2 overview*n5 pathway* or systematic n2 synthesis n5 pathway* or systematic n2 syntheses n5 pathway* or systematic n2 literature review* n5 pathway* or systematic n2 evidence review* n5 pathway* or systematic n2 review* n5 algorithm* or systematic n2 overview*n5 algorithm* or systematic n2 synthesis n5 algorithm* or systematic n2 syntheses n5 algorithm* or systematic n2 literature review* n5 algorithm* or systematic n2 evidence review* n5 algorithm*"" | 49 |
| S13 | ""systematic n2 review* n5 health care or systematic n2 overview*n5 health care or systematic n2 synthesis n5 health care or systematic n2 syntheses n5 health care or systematic n2 literature review* n5 health care or systematic n2 evidence review* n5 health care or systematic n2 review* n5 healthcare or systematic n2 overview*n5 healthcare or systematic n2 synthesis n5 healthcare or systematic n2 syntheses n5 healthcare or systematic n2 literature review* n5 healthcare or systematic n2 evidence review* n5 healthcare systematic n2 review* n5 health care or systematic n2 overview*n5 health care or systematic n2 synthesis n5 health care or systematic n2 syntheses n5 health care or systematic n2 literature review* n5 health care or systematic n2 evidence review* n5 health care or systematic n2 review* n5 healthcare or systematic n2 overview*n5 healthcare or systematic n2 synthesis n5 healthcare or systematic n2 syntheses n5 healthcare or systematic n2 literature review* n5 healthcare or systematic n2 evidence review* n5 health care"" | 421 |
| S12 | ""systematic n2 review* n5 patient care or systematic n2 overview*n5 patient care or systematic n2 synthesis n5 patient care or systematic n2 syntheses n5 patient care or systematic n2 literature review* n5 patient care or systematic n2 evidence review* n5 patient care or systematic n2 review* n5 patient care or systematic n2 overview*n5 patient care or systematic n2 synthesis n5 patient care or systematic n2 syntheses n5 patient care or systematic n2 literature review* n5 patient care or systematic n2 evidence review* n5 patient caresystematic n2 review* n5 patient care or systematic n2 overview*n5 patient care or systematic n2 synthesis n5 patient care or systematic n2 syntheses n5 patient care or systematic n2 literature review* n5 patient care or systematic n2 evidence review* n5 patient care or systematic n2 review* n5 patient care or systematic n2 overview*n5 patient care or systematic n2 synthesis n5 patient care or systematic n2 syntheses n5 patient care or systematic n2 literature review* n5 patient care or systematic n2 evidence review* n5 patient care"" | 213 |
| S11 | (systematic n2 review* n5 treatment plan* or systematic n2 overview*n5 treatment plan* or systematic n2 synthesis n5 treatment plan* or systematic n2 syntheses n5 treatment plan* or systematic n2 literature review* n5 treatment plan* or systematic n2 evidence review* n5 treatment plan* or systematic n2 review* n5 clinical decision* or systematic n2 overview*n5 clinical decision* or systematic n2 synthesis n5 hospital* decision* or systematic n2 syntheses n5 clinical decision* or systematic n2 literature review* n5 clinical decision* or systematic n2 evidence review* n5 clinical decision*systematic n2 review* n5 treatment plan* or systematic n2 overview*n5 treatment plan* or systematic n2 synthesis n5 treatment plan* or systematic n2 syntheses n5 treatment plan* or systematic n2 literature review* n5 treatment plan* or systematic n2 evidence review* n5 treatment plan* or systematic n2 review* n5 clinical decision* or systematic n2 overview*n5 clinical decision* or systematic n2 synthesis n5 hospital* decision* or systematic n2 syntheses n5 clinical decision* or systematic n2 literature review* n5 clinical decision*) | 37 |
| S10 | (systematic n2 review* n5 summar* or systematic n2 overview*n5 summar* or systematic n2 synthesis n5 summar* or systematic n2 syntheses n5 summar* or systematic n2 literature review* n5 summar* or systematic n2 evidence review* n5 summar* or systematic n2 review* n5 hospital* decision* or systematic n2 overview*n5 hospital* decision* or systematic n2 synthesis n5 hospital* decision* or systematic n2 syntheses n5 hospital* decision* or systematic n2 literature review* n5 hospital* decision* or systematic n2 evidence review* n5 hospital* decision*systematic n2 review* n5 summar* or systematic n2 overview*n5 summar* or systematic n2 synthesis n5 summar* or systematic n2 syntheses n5 summar* or systematic n2 literature review* n5 summar* or systematic n2 evidence review* n5 summar* or systematic n2 review* n5 hospital* decision* or systematic n2 overview*n5 hospital* decision* or systematic n2 synthesis n5 hospital* decision* or systematic n2 syntheses n5 hospital* decision* or systematic n2 literature review* n5 hospital* decision* or systematic n2 overview*n5 hospital* decision*) | 552 |
| S9 | (systematic n2 review* n5 utili* or systematic n2 overview*n5 utili* or systematic n2 synthesis n5 utili* or systematic n2 syntheses n5 utili* or systematic n2 literature review* n5 utili* or systematic n2 evidence review* n5 utili* or systematic n2 review* n5 disseminat* or systematic n2 overview*n5 disseminat* or systematic n2 synthesis n5 disseminat* or systematic n2 syntheses n5 disseminat* or systematic n2 literature review* n5 disseminat* or systematic n2 evidence review* n5 disseminat*) | 266 |
| S8 | (systematic n2 review* n5 application or systematic n2 overview*n5 application or systematic n2 synthesis n5 application or systematic n2 syntheses n5 application or systematic n2 literature review* n5 application or systematic n2 evidence review* n5 application or systematic n2 review* n5 implement* or systematic n2 overview*n5 implement* or systematic n2 synthesis n5 implement* or systematic n2 syntheses n5 implement* or systematic n2 literature review* n5 implement* or systematic n2 evidence review* n5 implement*systematic n2 review* n5 application or systematic n2 overview*n5 application or systematic n2 synthesis n5 application or systematic n2 syntheses n5 application or systematic n2 literature review* n5 application or systematic n2 evidence review* n5 application or systematic n2 review* n5 implement* or systematic n2 overview*n5 implement* or systematic n2 synthesis n5 implement* or systematic n2 syntheses n5 implement* or systematic n2 literature review* n5 implement* or systematic n2 evidence review* n5 implement*systematic n2 review* n5 application) | 179 |
| S7 | (systematic n2 review* n5 action* or systematic n2 overview*n5 action* or systematic n2 synthesis n5 action* or systematic n2 syntheses n5 action* or systematic n2 literature review* n5 action* or systematic n2 evidence review* n5 action* or systematic n2 review* n5 practice or systematic n2 overview*n5 practice or systematic n2 synthesis n5 practice or systematic n2 syntheses n5 practice or systematic n2 literature review* n5 practice or systematic n2 evidence review* n5 practice) | 698 |
| S6 | (systematic n2 review* n5 budgethold* or systematic n2 overview*n5 budgethold* or systematic n2 synthesis n5 budgethold* or systematic n2 syntheses n5 budgethold* or systematic n2 literature review* n5 budgethold* or systematic n2 evidence review* n5 budgethold* or systematic n2 review* n5 budget hold* or systematic n2 overview*n5 budget hold* or systematic n2 synthesis n5 budget hold* or systematic n2 syntheses n5 budget hold* or systematic n2 literature review* n5 budget hold* or systematic n2 evidence review* n5 budget hold*systematic n2 review* n5 budgethold* or systematic n2 overview*n5 budgethold* or systematic n2 synthesis n5 budgethold* or systematic n2 syntheses n5 budgethold* or systematic n2 literature review* n5 budgethold* or systematic n2 evidence review* n5 budgethold* or systematic n2 review* n5 budget hold* or systematic n2 overview*n5 budget hold* or systematic n2 synthesis n5 budget hold* or systematic n2 syntheses n5 budget hold* or systematic n2 literature review* n5 budget hold* or systematic n2 synthesis n5 budgethold*) | 0 |
| S5 | (systematic n2 review* n5 procur* or systematic n2 overview*n5 procur* or systematic n2 synthesis n5 procur* or systematic n2 syntheses n5 procur* or systematic n2 literature review* n5 procur* or systematic n2 evidence review* n5 procur* or systematic n2 review* n5 service provi* or systematic n2 overview*n5 service provi* or systematic n2 synthesis n5 service provi* or systematic n2 syntheses n5 service provi* or systematic n2 literature review* n5 service provi* or systematic n2 evidence review* n5 service provi*systematic n2 review* n5 procur* or systematic n2 overview*n5 procur* or systematic n2 synthesis n5 procur* or systematic n2 syntheses n5 procur* or systematic n2 literature review* n5 procur* or systematic n2 evidence review* n5 procur* or systematic n2 review* n5 service provi* or systematic n2 overview*n5 service provi* or systematic n2 synthesis n5 service provi* or systematic n2 syntheses n5 service provi* or systematic n2 literature review* n5 service provi* or systematic n2 evidence review* n5 service provi*systematic n2 review* n5 procur*) | 19 |
| S4 | (systematic n2 review* n5 commission* or systematic n2 overview*n5 commission* or systematic n2 synthesis n5 commission* or systematic n2 syntheses n5 commission* or systematic n2 literature review* n5 commission* or systematic n2 evidence review* n5 commission* or systematic n2 review* n5 purchas* or systematic n2 overview*n5 purchas* or systematic n2 synthesis n5 purchas* or systematic n2 syntheses n5 purchas* or systematic n2 literature review* n5 purchas* or systematic n2 evidence review* n5 purchas*systematic n2 review* n5 commission* or systematic n2 overview*n5 commission* or systematic n2 synthesis n5 commission* or systematic n2 syntheses n5 commission* or systematic n2 literature review* n5 commission* or systematic n2 evidence review* n5 commission* or systematic n2 review* n5 purchas* or systematic n2 overview*n5 purchas* or systematic n2 synthesis n5 purchas* or systematic n2 syntheses n5 purchas* or systematic n2 literature review* n5 purchas* or systematic n2 evidence review* n5 purchas*systematic n2 review* n5 commission*) | 44 |
| S3 | (systematic n2 review* n5 policy decision* or systematic n2 overview*n5 policy decision* or systematic n2 synthesis n5 policy decision* or systematic n2 syntheses n5 policy decision* or systematic n2 literature review* n5 policy decision* or systematic n2 evidence review* n5 policy decision* or systematic n2 review* n5 health manage* or systematic n2 overview*n5 health manage* or systematic n2 synthesis n5 health manage* or systematic n2 syntheses n5 health manage* or systematic n2 literature review* n5 health manage* or systematic n2 evidence review* n5 health manage*systematic n2 review* n5 policy decision* or systematic n2 overview*n5 policy decision* or systematic n2 synthesis n5 policy decision* or systematic n2 syntheses n5 policy decision* or systematic n2 literature review* n5 policy decision* or systematic n2 evidence review* n5 policy decision* or systematic n2 review* n5 health manage* or systematic n2 overview*n5 health manage* or systematic n2 synthesis n5 health manage* or systematic n2 syntheses n5 health manage* or systematic n2 literature review* n5 policy decision*) | 40 |
| S2 | ""S2 systematic n2 review* n5 policy making or systematic n2 overview*n5 policy making or systematic n2 synthesis n5 policy making or systematic n2 syntheses n5 policy making or systematic n2 literature review* n5 policy making or systematic n2 evidence review* n5 policy making or systematic n2 review* n5 policy making or systematic n2 overview*n5 policy making or systematic n2 synthesis n5 policy making or systematic n2 syntheses n5 policy making or systematic n2 literature review* n5 policy making or systematic n2 evidence review* n5 policy makingsystematic n2 review* n5 policy making or systematic n2 overview*n5 policy making or systematic n2 synthesis n5 policy making or systematic n2 syntheses n5 policy making or systematic n2 literature review* n5 policy making or systematic n2 evidence review* n5 policy making or systematic n2 review* n5 policy making or systematic n2 overview*n5 policy making or systematic n2 synthesis n5 policy making or systematic n2 syntheses n5 policy making or systematic n2 literature review* n5 policy making or systematic n2 synthesis n5 policy making"" | 7 |
| S1 | ""systematic n2 review* n5 decision making or systematic n2 overview*n5 decision making or systematic n2 synthesis n5 decision making or systematic n2 syntheses n5 decision making or systematic n2 literature review* n5 decision making or systematic n2 evidence review* n5 decision making or systematic n2 review* n5 decision-making or systematic n2 overview*n5 decision-making or systematic n2 synthesis n5 decision-making or systematic n2 syntheses n5 decision-making or systematic n2 literature review* n5 decision-making or systematic n2 evidence review* n5 decision-makingsystematic n2 review* n5 decision making or systematic n2 overview*n5 decision making or systematic n2 synthesis n5 decision making or systematic n2 syntheses n5 decision making or systematic n2 literature review* n5 decision making or systematic n2 evidence review* n5 decision making or systematic n2 review* n5 decision-making or systematic n2 overview*n5 decision-making or systematic n2 synthesis n5 decision-making or systematic n2 syntheses n5 decision-making or systematic n2 literature review* n5 decision making"" | 105 |

**Online supplement 2: Grey Literature Sources**

3ie Policy Briefs (<http://www.3ieimpact.org/en/evidence/policy-briefs/>)

Canadian Agency for Drugs and Technology in Health (<https://www.cadth.ca/>)

Capacity Plus (<http://www.capacityplus.org/>)

CDC Community Guide (<http://www.thecommunityguide.org/index.html>)

Communicate to Vaccinate (<http://www.commvac.com/>)

Consortium for Research on Equitable Health Systems (<http://www.crehs.lshtm.ac.uk/>)

Developing and Evaluating Communication Strategies to Support Informed Decisions and Practice Based on Evidence (<http://www.decide-collaboration.eu/>)

Epistemonikos (<http://www.epistemonikos.org/>)

Evidence Aid (<http://www.evidenceaid.org/>)

EVIPNet/SURE (<http://global.evipnet.org/en>) and (<http://www.who.int/evidence/sure/policybriefs/en/>)

Global HIV/AIDS Initiatives Network (GHIN) (<http://www.aidsmap.com/>)

Health Action International (<http://haiweb.org/>)

Health Systems Evidence (<https://www.healthsystemsevidence.org/>)

Human Sciences Research Council (<http://www.hsrc.ac.za/en>)

IntraHealth International/Capacity Project (<http://www.intrahealth.org/page/capacityplus>)

McMaster Health Forum Evidence briefs (<https://www.mcmasterhealthforum.org/>)

Partnership for maternal, newborn, and child health (WHO) (<http://www.who.int/pmnch/en/>)

PDQ evidence (<http://www.pdq-evidence.org/>)

Rx for change (<http://rxforchange.ucsf.edu/>)

Social, Technological and Environmental Pathways to Sustainability Centre (<http://steps-centre.org/#&panel1-1>)

SUPPORT Summaries (<http://supportsummaries.org/>)

WHO - Department of Health Systems Financing (<http://www.who.int/healthsystems/topics/financing/en/>)

WHO - Department of human resources for health (<http://www.who.int/hrh/about/en/> OR <http://www.who.int/hrh/en/>)

World Bank - Reaching the poor (<https://openknowledge.worldbank.org/handle/10986/7393>)

**Online supplement 3: Excluded studies**

| **Ref ID** | **Reason for Exclusion** |
| --- | --- |
| Alper 2005 | Did not include policymakers |
| Bartels 2011 | Did not include policymakers |
| Bero 1997 | The intervention did not assess summaries/derivatives of systematic reviews |
| Caruana 2008 | Not an eligible study design, did not include policymakers |
| Chambers 2011 | Not an eligible study design – scoping review |
| Chambers 2012 | Not an eligible study design - description of the preparation and evaluation of an evidence brief |
| Coulter 2006 | Not an eligible study design – criteria for evaluating quality of evidence summary and synthesis |
| Dobbins 2001 | Not an eligible study design – Cross-sectional survey on the use of 5 complete systematic reviews (not derivative products) |
| Dobbins 2004 | Intervention included complete systematic review not a derivative product. |
| Dobbins 2007 | Not an eligible study design – Qualitative study assessing participant definitions of evidence-based decision-making and preferences for receiving research information |
| Fahey 1995 | Wrong intervention – compared 4 ways of presenting effect sizes in RCT or systematic review |
| Kelechi 2010 | Not an eligible study design – steps for appraising evidence |
| Kendall 2013 | Not an eligible study design - editorial |
| Kirkpatrick 1995 | Did not include policymakers |
| Lavis 2005a | Not an eligible study design – systematic review |
| Lavis 2011 | RCT was not conducted because of limited enrollment. The authors instead conducted interviews so this was not an eligible study design. |
| Lorenc 2014 | Not an eligible study design – systematic review |
| MacFarlane 2011 | Not an eligible study design – qualitative evaluation |
| MacGregor 2014 | Not an eligible study design – systematic review |
| Madhavan 2012 | Not an eligible study design – narrative review of 4 recommendations for evidence−based management |
| Mallory 2010 | Not an eligible study design – narrative review to describe evidence-based practice strategies for nursing societies |
| Malterud 2016 | Not an eligible study design – case study exploring publications from the Norwegian Knowledge Centre for the Health Services |
| Maluka 2014 | Not an eligible study design – no control group, describes a research project that aimed to implement an accountability for reasonableness approach to priority setting in decision-making |
| Mitchell 2011 | Not an eligible study design – description of systematic review and literature review |
| Moat 2013 | Not an eligible study design – systematic review |
| Mossialos 2013 | Not an eligible study design – overview of systematic reviews |
| Munn 2015 | Not an eligible study design – describes two forms of evidence nventory reports |
| Murthy 2012 | Not an eligible study design- systematic review |
| Nannini 2010 | Not an eligible study design – narrative review of the role of gerontological nurses in translating systematic review evidence |
| Noor 2009 | Not an eligible study design – narrative review of health research oversight structures and systems available in Africa |
| Nutley 2014 | Intervention did not include systematic review derivatives |
| Oermann 2009 | Did not include policy makers |
| Oliver 2014 | Not an eligible study design- systematic review |
| Perrier 2011a | Not an eligible study design- systematic review |
| Perrier 2011b | Not an eligible study design- systematic review |
| Perrier 2015 | Did not include policy makers |
| Rosenbaum 2011 | Not an eligible study design – user testing, interviews, no control group |
| Santesso 2015 | Did not include policy makers (patients and the public) |
| Sullivan 2014 | Not an eligible study design - systematic review |
| Taylor-Robinson 2008 | Not an eligible study design - qualitative |
| Thomson 2013a | Not an eligible study design – narrative review |
| Thomson 2013b | Not an eligible study design – describes methods of preparing visual summary |
| Tricco 2016 | Not an eligible study design - Scoping review |
| Wallace 2014 | Not an eligible study design - Systematic review |
| Wilson 2013 | Not an eligible study design – assessment of the types of evidence within the Health Systems Evidence database |
| Yavchitz 2014 | Did not include policy makers |

**Online supplement 4: Risk of bias assessments**

**Brownson 2011**

| **Bias** | **Authors’ Judgement** | **Support for judgement** |
| --- | --- | --- |
| Random sequence generation | Unclear | Not reported |
| Allocation Concealment | Unclear | Not reported |
| Baseline outcome measurement | Unclear | Not reported |
| Baseline characteristics similar | Low risk of bias | No study condition differences by age, sex, self-reported health status, education level, fiscal or social position |
| Incomplete outcome data | High risk of bias | Low response rate |
| Knowledge of allocated intervention | Unclear | Not reported |
| Adequate protection from contamination | Unclear | Not reported |
| Selective outcome reporting | Low risk of bias | Reports all stated outcomes |
| Other risks of bias | Low risk of bias | None detected |

**Carrasco-Labra 2016**

| **Bias** | **Authors’ Judgement** | **Support for judgement** |
| --- | --- | --- |
| Random sequence generation | Low risk of bias | Randomization scheme automatically generated by SurveyMonkey |
| Allocation Concealment | Low risk of bias | Allocation done by survey monkey in real time following an algoritm umknown to the authors |
| Baseline outcome measurement | Unclear | Not reported |
| Baseline characteristics similar | Unclear | Some differences but not clear if they are significant - e.g. more women in control, more French, Spanish, Italian native speakers in new group, more clinicians in new group, more researchers in control |
| Incomplete outcome data | Low risk of bias | All participants analyzed for whom the variables of interest were present |
| Knowledge of allocated intervention | Low risk of bias | To conceal the nature of the Summary of Findings tables to which participants were allocated, the tables were labeled as A or B, without any other information about their content or the study hypothesis. |
| Adequate protection from contamination | Low risk of bias | The randomization scheme was automatically generated by the platform. When direct comparison between the new and current format was required, the order in which the tables were shown to participants was randomly determined. To conceal the nature of the Summary of Findings tables to which participants were allocated, the tables were labeled as A or B, without any other information about their content or the study hypothesis. |
| Selective outcome reporting | Low risk of bias | Protocol specifies all outcomes assessed |
| Other risks of bias | Low risk of bias | None detected |

**Dobbins 2009**

| **Bias** | **Authors’ Judgement** | **Support for judgement** |
| --- | --- | --- |
| Random sequence generation | Low risk of bias | Randomly allocated to groups using computer-generated random numbers |
| Allocation Concealment | Unclear | Not reported |
| Baseline outcome measurement | Low risk of bias | No statistically significant differences were observed between groups at baseline |
| Baseline characteristics similar | Unclear | Not reported |
| Incomplete outcome data | Low risk of bias | Follow up data for 88 or 108 PHD - reasons given were lack of time, not having someone working on health weight promotion for children, similar drop outs for all groups |
| Knowledge of allocated intervention | Unclear | Not reported |
| Adequate protection from contamination | Low risk of bias | All Health departments in CAN were invited to participate and were identified through provincial databases. |
| Selective outcome reporting | Low risk of bias | Assessed all outcome outlined as important |
| Other risks of bias | Low risk of bias | Appears to be free of other risks of bias |

**Masset 2013**

| **Bias** | **Authors’ Judgement** | **Support for judgement** |
| --- | --- | --- |
| Random sequence generation | Unclear | Not reported |
| Allocation Concealment | Unclear | Not reported |
| Baseline outcome measurement | Low risk of bias | No differences |
| Baseline characteristics similar | Low risk of bias | Participants were equally divided by gender and by residence in a high income country. There were no differences in characteristics. |
| Incomplete outcome data | High risk of bias | High attrition (Only 50 per cent of the original sample participated in the first follow-up, a further 36 per cent dropped out at the 1-week follow-up and a further 11 per cent dropped out before the 3-month follow-up) |
| Knowledge of allocated intervention | Unclear | Not reported |
| Adequate protection from contamination | Unclear | Not reported |
| Selective outcome reporting | Low risk of bias | Seems to report all planned. |
| Other risks of bias | Unclear | High attrition may generate two types of biases. The first bias arises from self-selection of respondents into the survey, while the second bias arises from differential attrition among survey groups during the survey rounds |

**Opiyo 2013**

| **Bias** | **Authors’ Judgement** | **Support for judgement** |
| --- | --- | --- |
| Random sequence generation | Unclear | Not reported |
| Allocation Concealment | Unclear | Not reported |
| Baseline outcome measurement | Unclear | Not reported |
| Baseline characteristics similar | Unclear | Not reported |
| Incomplete outcome data | Low risk of bias | 7 didn’t attend workshop, 5 didn't complete questionnaires - The most common reason for non-attendance was related to timing of the meeting |
| Knowledge of allocated intervention | Unclear | Not reported |
| Adequate protection from contamination | Unclear | Not reported |
| Selective outcome reporting | Low risk of bias | Seems to report all outcomes |
| Other risks of bias | Low risk of bias | None detected |

**Vandvik 2012**

| **Bias** | **Authors’ Judgement** | **Support for judgement** |
| --- | --- | --- |
| Random sequence generation | Unclear | Not reported |
| Allocation Concealment | Low risk of bias | "There was no need to conceal allocation as the randomization procedure was performed in one sequence before start of the study" |
| Baseline outcome measurement | Unclear | Not reported |
| Baseline characteristics similar | Unclear | Not reported |
| Incomplete outcome data | Low risk of bias | All those randomized completed the study |
| Knowledge of allocated intervention | Low risk of bias | Panelists blinded, data collection monitored by blinded investigators, data analysis of results was blinded |
| Adequate protection from contamination | Unclear | Not reported |
| Selective outcome reporting | Low risk of bias | Seems to report all outcomes |
| Other risks of bias | Low risk of bias | None detected |
